# Glycoprotein In Vitro N-Glycan Processing Using Enzymes Expressed in *E. coli*

**DOI:** 10.3390/molecules28062753

**Published:** 2023-03-18

**Authors:** Libo Zhang, Yanhong Li, Riyao Li, Xiaohong Yang, Zimin Zheng, Jingxin Fu, Hai Yu, Xi Chen

**Affiliations:** Department of Chemistry, University of California, Davis, CA 95616, USA

**Keywords:** N-glycan, glycoprotein, glycan engineering, glycosyltransferase, mannosidase, *E. coli* expression

## Abstract

Protein N-glycosylation is a common post-translational modification that plays significant roles on the structure, property, and function of glycoproteins. Due to N-glycan heterogeneity of naturally occurring glycoproteins, the functions of specific N-glycans on a particular glycoprotein are not always clear. Glycoprotein in vitro N-glycan engineering using purified recombinant enzymes is an attractive strategy to produce glycoproteins with homogeneous N-glycoforms to elucidate the specific functions of N-glycans and develop better glycoprotein therapeutics. Toward this goal, we have successfully expressed in *E. coli* glycoside hydrolases and glycosyltransferases from bacterial and human origins and developed a robust enzymatic platform for in vitro processing glycoprotein N-glycans from high-mannose-type to α2–6- or α2–3-disialylated biantennary complex type. The recombinant enzymes are highly efficient in step-wise or one-pot reactions. The platform can find broad applications in N-glycan engineering of therapeutic glycoproteins.

## 1. Introduction

Protein N-glycosylation is an important post-translational modification that affects the structure, property, and function of glycoproteins including folding, solubility, stability, localization, trafficking, molecular recognition, and interactions, etc. Glycoprotein N-glycans are attached via the innermost *N*-acetylglucosamine residue to the L-asparagine residue (GlcNAcβ1–Asn) in the Asn-X-Ser/Thr sequon (where X is an amino acid that is not an L-proline) of the protein with a β-linked N-glycosidic bond. All eukaryotic glycoprotein N-glycans share a trimannosyl chitobiose (Man_3_GlcNAc_2_) core and can be classified as high-mannose, complex, and hybrid types based on the glycan structures extended from the terminal mannose residues on the core [1,2].

Many therapeutic proteins and enzymes are N-glycosylated. The level of N-glycosylation and the structure of their N-glycans can directly affect their solubility, stability, safety, function, efficacy, delivery, pharmacokinetics, immunogenicity, and dose frequency [1,3,4,5,6,7,8,9]. Therefore, N-glycosylation is a critical quality attribute (CQA) of glycoprotein therapeutics considered by regulatory authorities [3,5,10]. Homogeneous glycoproteins with preferred N-glycoforms are highly desirable for their pharmaceutical applications and for exploring the fundamental understanding of their functions at the molecular level [7].

Nevertheless, N-glycosylated glycoproteins are intrinsically heterogeneous with variations on the N-glycan location, site occupancy of N-glycosylation, and N-glycan structures at individual N-glycosylation sites [11,12,13], and the N-glycosylation process is influenced by many factors [3,14]. Cells of different origins have been used to produce N-glycosylated glycoproteins [4]. Numerous strategies including cell line engineering, as well as the addition of inhibitors for metabolic glycosylation and glycosidase have been developed to reduce glycoprotein N-glycan heterogeneity [15,16]. Synthetic methods including total synthesis, semi-synthesis, and chemoenzymatic synthesis have been developed to obtain homogeneous glycoproteins [17,18,19,20]. It is, however, still challenging to achieve glycoprotein N-glycan homogeneity [15], especially in large-scale productions. For example, monoclonal IgG1-type antibodies are an important type of therapeutic glycoproteins that have seen significant increased clinical applications. The desired N-glycans at the conserved N-glycosylation site of the Fc domain of these antibodies are biantennary complex-type fully sialylated with α2–6-linked *N*-acetylneuraminic acid (Neu5Ac) without a core fucose [21]. Nevertheless, commercial therapeutic monoclonal antibodies have undesired high levels of high mannose-type N-glycans that are challenging to control during manufacturing processes [22]. In vitro enzymatic conversion of the high-mannose-type N-glycans on the glycoproteins to the preferred complex biantennary-type is a potential solution to overcome the challenge.

Among the methods developed for producing glycoproteins with structurally defined homogeneous N-glycans [4,19], the strategy of using recombinant glycoside hydrolases and glycosyltransferases for in vitro modifying N-glycan of glycoproteins is attractive [16,23]. Success in kilogram-scale N-glycan engineering of a recombinant human immunoglobulin G (hIgG) antibody using a purified recombinant β1–4-galactosyltransferase 1 (Bβ4GalT1) [24] highlights the feasibility of applying enzymatic glycoprotein in vitro N-glycan processing for large-scale production of therapeutic glycoproteins with structurally defined N-glycans. Nevertheless, the scope of several reported examples [25,26,27,28] is limited to the involvement of one or two glycosyltransferases with or without glycoside hydrolases. The utilization of multiple carbohydrate-active enzymes including glycoside hydrolases and glycosyltransferases for glycoprotein in vitro N-glycan processing has been traditionally underexplored, but has shown a high potential in several recent examples [23,29]. In general, glycosyltransferases that are expressed in mammalian [24,27] or insect cells [25] have been commonly used for glycoprotein N-glycan engineering due to the challenges in obtaining their active forms from common *E. coli* expression systems [29,30]. As many of these enzymes expressed in eukaryotic cells are N-glycosylated glycoproteins themselves, their N-glycans may complicate N-glycan analysis of target glycoproteins, a potential problem that deserves consideration and investigation, but has not been paid sufficient attention.

We believe that glycoprotein in vitro N-glycan processing and engineering using purified recombinant enzymes expressed in *E. coli* is an efficient platform that can be combined conveniently with different glycoprotein production systems to produce glycoproteins with homogeneous N-glycoforms. Using enzymes expressed in *E. coli* has the advantage of not introducing external N-glycans that may interfere with N-glycan analysis of target glycoproteins. *E. coli* expression system is also considered as the most convenient and economical system for protein production [29,31].

Using ribonuclease B (RNase B), a glycoprotein with high-mannose-type N-glycans at a single N-glycosylation site [32,33], as a model glycoprotein, we demonstrate here the success of processing glycoprotein N-glycans from high-mannose type to α2–3 or α2–6-linked disialylated complex biantennary N-glycans, using recombinant enzymes expressed in *E. coli* in both step-wise and one-pot reactions.

## 2. Results and Discussion

### 2.1. Glycoprotein In Vitro N-Glycan Processing Route Design and Enzyme Selection

**Figure 1:**
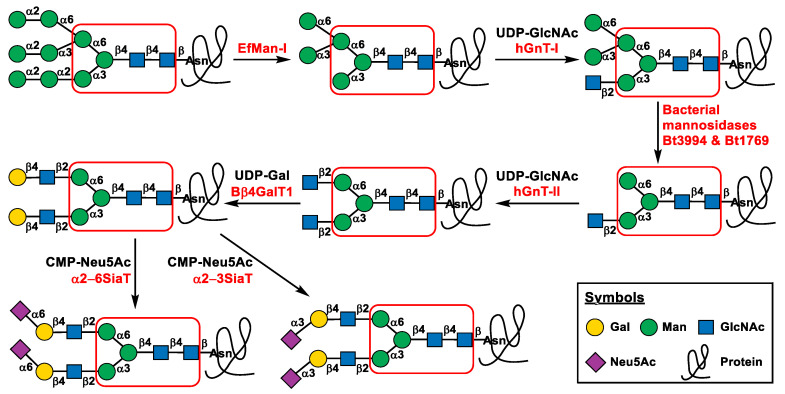
Glycoprotein in vitro N-glycan processing from high-mannose to disialylated biantennary complex-type N-glycans. The conserved trimannosyl chitobiose core (Man_3_GlcNAc_2_) of eukaryotic glycoprotein N-glycans is highlighted in a red rectangle.

To establish an efficient platform for processing glycoprotein N-glycans from high-mannose-type to disialylated complex biantennary N-glycans, commercially available bovine pancreatic RNase B was chosen as a model. It is a relatively small glycoprotein of 124 amino acids with a single N-glycosylation site at Asn34, which is attached with high mannose-type N-glycans (Man_5-9_GlcNAc_2_) containing five to nine mannose residues [32,33,34]. As eukaryotic glycosyltransferases involved in N-glycan processing are well known and the corresponding bacterial alternatives have not been fully identified, attempts to express the former in *E. coli* are one of the focuses of our enzymatic glycoprotein in vitro N-glycan processing strategy. Meanwhile, bacterial alternatives of eukaryotic N-glycan processing mannosidases and glycosyltransferases that have already been identified with desired functions are great choices for developing the efficient glycoprotein in vitro N-glycan processing platform. As shown in Figure 1, the heterogeneous Man_5-9_GlcNAc_2_ N-glycans on RNase B can be processed to a homogeneous Man_5_GlcNAc_2_ N-glycan by removing all α1–2-linked mannose residues, using a recombinant *Enterococcus faecalis* α1–2-mannosidase (EfMan-I) expressed in *E. coli* that we reported previously [34]. EfMan-I belongs to the carbohydrate active enzyme (CAZy) glycoside hydrolase [35,36] family 92 (GH92) and requires a divalent metal cation, such as Ca^2+^ or Mg^2+^ for activity [34]. After EfMan-I treatment, the natural N-glycan processing steps [2] can be followed in vitro using recombinant glycosyltransferases and glycosidases from different origins to form target homogeneous glycoproteins with the desired disialylated biantennary complex-type N-glycans.

Human N-acetylglucosaminyltransferase I (hGnT-I or hMGAT1) in the CAZy glycosyltransferase [37,38] family 13 (GT13) was chosen to add an *N*-acetylglucosamine (GlcNAc) residue β1–2-linked to the α1–3-linked mannose residue on the trimannosyl chitobiose core of the Man_5_GlcNAc_2_ N-glycan on the EfMan-I-treated RNase B to form a hybrid-type GlcNAcMan_5_GlcNAc_2_ N-glycan. The hGnT-I uses uridine-5′-diphosphate GlcNAc (UDP-GlcNAc) as the donor substrate and requires a divalent metal cation, such as Mn^2+^ as a cofactor. It is highly selective toward Man_5_GlcNAc_2_ N-glycan with dramatically decreased activity for Man_3_GlcNAc_2_ and other high-mannose-type N-glycans [39]. This acceptor substrate preference is beneficial for our one-pot multienzyme (OPME) N-glycan processing approach described below.

To process the GlcNAcMan_5_GlcNAc_2_ N-glycan on glycoproteins further to form GlcNAcMan_3_GlcNAc_2_, the reported *Bacteroides thetaiotaomicron* α1–6-mannosidase Bt3994 and α1–3-mannosidase Bt1769 [40] were chosen. Similar to EfMan-I, they are Ca^2+^-dependent CAZy GH92 bacterial mannosidases [40]. In contrast to EfMan-I, which is an α1–2-mannosidase, Bt3994 was reported as an α1–6-mannosidase to catalyze the cleavage of the terminal α1–6-mannosidic linkage in Man_5_GlcNAc_2_ and Man_3_GlcNAc_2_ N-glycans to form Man_4_GlcNAc_2_ and Man_2_GlcNAc_2_, respectively, while Bt1769 was reported as an α1–3-mannosidase to catalyze the cleavage of the terminal unbranched α1–3-linked mannose of the Man_4_GlcNAc_2_ N-glycan generated by Bt3994 to form Man_3_GlcNAc_2_ [40]. Based on the reported activities of Bt3994 and Bt1769, we hypothesized that they can coordinate with each other to convert GlcNAcMan_5_GlcNAc_2_ N-glycan on glycoproteins to GlcNAcMan_3_GlcNAc_2_.

To generate the second antenna in the glycoprotein biantennary complex N-glycans, human N-acetylglucosaminyltransferase II (hGnT-II or hMGAT2, CAZy family GT16) was chosen. It requires a divalent metal cation for catalyzing the transfer of GlcNAc from UDP-GlcNAc to form a β1–2-linkage to the α1–6-linked terminal mannose on GlcNAcMan_3_GlcNAc_2_ to produce GlcNAc_2_Man_3_GlcNAc_2_. hGnT-II has been shown to have a substrate binding pocket that interacts with both the α1–6-linked terminal mannose as the glycosylation site and the other GlcNAcβ1–2Manα1–3Manβ branch as the additional “recognition arm” [41]. This high acceptor substrate selectivity, again, is advantageous for the OPME N-glycan engineering approach presented below.

The biantennary GlcNAc_2_Man_3_GlcNAc_2_ complex-type N-glycan on RNase B generated from the hGnT-II reaction can be β1–4-galactosylated using a well-known bovine β1–4-galactosyltransferase 1 (Bβ4GalT1) [42,43,44,45], a CAZy GT7 family enzyme, to form RNase B containing Gal_2_GlcNAc_2_Man_3_GlcNAc_2_ N-glycan using uridine 5′-diphosphate galactose (UDP-Gal) as the donor substrate.

The in vitro N-glycan processing can be completed by a final sialylation step using a suitable sialyltransferase in the presence of CMP-sialic acid to form RNase B containing homogeneous α2–3- or α2–6-sialylated biantennary complex-type N-glycans (Sia_2_Gal_2_GlcNAc_2_Man_3_GlcNAc_2_). Different sialic acid forms can be introduced in the enzymatic sialylation step [46] and the most common sialic acid form, *N*-acetylneuraminic acid (Neu5Ac), is introduced as an example in our study presented here.

### 2.2. Enzyme Cloning and Expression

To facilitate the purification of enzymes that will be used for glycoprotein in vitro N-glycan processing, an His_6_-tag was introduced at the C-terminus of each recombinant enzyme to allow its easy purification by Ni^2+^-affinity columns. Furthermore, we found that fusing a maltose binding protein (MBP) at the N-terminus of the target recombinant protein and removing the N-terminal transmembrane domain of mammalian glycosyltransferases by truncation worked well to improve their soluble expression in *E. coli* [47]. These were the strategies that guided our general design to construct the plasmids for expressing target recombinant enzymes. In addition, *E. coli* Origami B (DE3) strain harboring pGro7 for chaperon expression was found to be a better choice for expressing mammalian enzymes than *E. coli* BL21 (DE3) strain, which was used to express recombinant enzymes from bacterial origins.

As we reported previously [34], EfMan-I was expressed as a C-terminal His_6_-tagged soluble and active enzyme in *E. coli* BL21 (DE3) cells with an expression level of 85 mg/L LB culture (Table 1).

**Figure 2:**
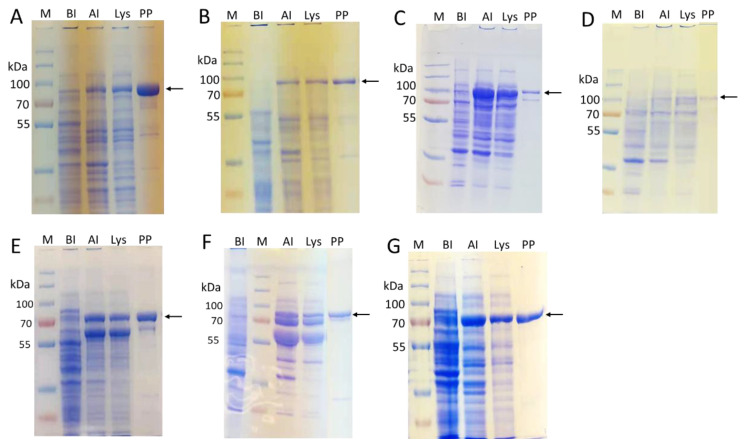
SDS-PAGE analysis results for enzyme expression and purification. (**A**) ∆24Bt3994-His_6_, (**B**) ∆18Bt1769-His_6_, (**C**) MBP-∆28hGnT-I-His_6_, (**D**) MBP-∆27hGnT-II-His_6_, (**E**) MBP-∆128Bβ4GalT1-His_6_, (**F**) MBP-∆89ST6GAL-I-His_6_, (**G**) MBP-CjCst-I∆145-His_6_. Lanes: BI, before induction; AI, after induction; Lys, lysate; PP, purified protein; M, PageRuler™ Plus Prestained Protein Ladder, 10 to 250 kDa. Target proteins are marked with an arrow on the right.

Although *Bacteroides thetaiotaomicron* mannosidases Bt3994 and Bt1769 were cloned previously in pET21a vector and expressed in *E. coli* Turner or B834 cells as C-terminal His_6_-tagged recombinant proteins [40], their expression levels were not reported. We found that removing Bt3994 N-terminal 24 amino acid residues and Bt1769 N-terminal 18 amino acid residues significantly improved their soluble expression levels in *E. coli* BL21 (DE3) cells. Both Δ24Bt3994-His_6_ and Δ18Bt1769-His_6_ were expressed at a level of around 55 mg/L LB media (Table 1) as soluble and active proteins with the expected molecular weights of 82 and 83 kDa, respectively (Figure 2A,B).

To obtain active and soluble recombinant hGnT-I and hGnT-II from *E. coli*, their N-terminal sequences containing the putative transmembrane domains were removed, and the truncated sequences were expressed as fusion proteins with an N-terminal maltose binding protein (MBP) and a C-terminal His_6_-tag. The resulting MBP-∆28hGnT-I-His_6_ and MBP-∆27hGnT-II-His_6_ were expressed at a level of 5 and 1 mg/L, respectively (Table 1), with the expected molecular weights of 91 and 92 kDa (Figure 2C,D).

Bβ4GalT1 expressed in *E. coli* has been purified from inclusion bodies [42,44,48]. We previously cloned and expressed an N-terminal 128 amino acid-truncated Bβ4GalT1 in pET15b as an N-terminal His_6_-tagged fusion protein (His_6_-∆128Bβ4GalT1) in *E. coli* BL21 (DE3) cells, which had a relatively low soluble expression (<1 mg/L culture) [45]. We redesigned the construct to express a protein with both an N-terminal MBP fusion and C-terminal His_6_-tag. The resulting MBP-∆128Bβ4GalT1-His_6_ was expressed in a dramatically improved 60 mg/L yield (Table 1) with an expected molecular weight at 75 kDa (Figure 2E).

For sialyltransferases, in addition to the recombinant sialyltransferases that we previously expressed in *E. coli* including bacterial α2–3-sialyltransferases, such as *Pasteurella multocida* α2–3-sialyltransferase 1 (PmST1) [49] and its mutant PmST1_M144D [50], *Pasteurella multocida* α2–3-sialyltransferase 3 (PmST3) [51], as well as α2–6-sialyltransferases, such as *Photobacterium damselae* α2–6-sialyltransfearse (Pd2,6ST) [52] and its mutant Pd2,6ST_A200Y/S232Y [53], *Photobacterium* species α2–6-sialyltransfearse (Psp2,6ST) [54] and its mutant Psp2,6ST_A366G [55], we cloned and expressed a recombinant human α2–6-sialyltransferase hST6GAL-I and a recombinant *Campylobacter jejuni* α2–3-sialyltransferase CjCst-I.

Similar to other eukaryotic sialyltransferases, hST6GAL-I is a CAZy GT29 enzyme [56]. It has been shown to selectively α2–6-sialylate glycoprotein N-glycans [57] and has been successfully expressed in *E. coli* as a soluble and active fusion protein with MBP at its N-terminus [31]. The soluble expression of the N-terminal MBP-fused and C-terminal His_6_-tagged N-terminal truncated hST6GAL-I (MBP-∆89hST6GAL-I-His_6_) that we constructed reached 30 mg/L LB culture (Table 1) with an expected molecular weight at 80 kDa (Figure 2F).

*Campylobacter jejuni* CjCst-I is an α2–3-sialyltransferase belonging to CAZy GT42 family [58,59]. It has been shown to utilize Galβ1–3/4OR as acceptors. The 145 residues at the C terminus of CjCst-I were removed and MBP was fused at the N terminus to form MBP-CjCst-I∆145-His_6_. It was successfully expressed in *E. coli* as a soluble and active fusion protein at a level of 60 mg/L LB culture with an expected molecular weight at 77 kDa (Figure 2G).

### 2.3. Step-Wise Reactions and Enzyme Activty Determination Using Glycoprotein Substrates

With all enzymes in hand, their activities and applications for glycoprotein in vitro N-glycan processing were tested using step-wise enzymatic reactions and the product formation was monitored by matrix-assisted laser desorption/ionization-time of flight (MALDI-TOF) mass spectrometry (MS) analysis. Reaction conditions were optimized by varying the types of the buffers used, pH, temperature, ion strength, incubation time, etc.

**Figure 3:**
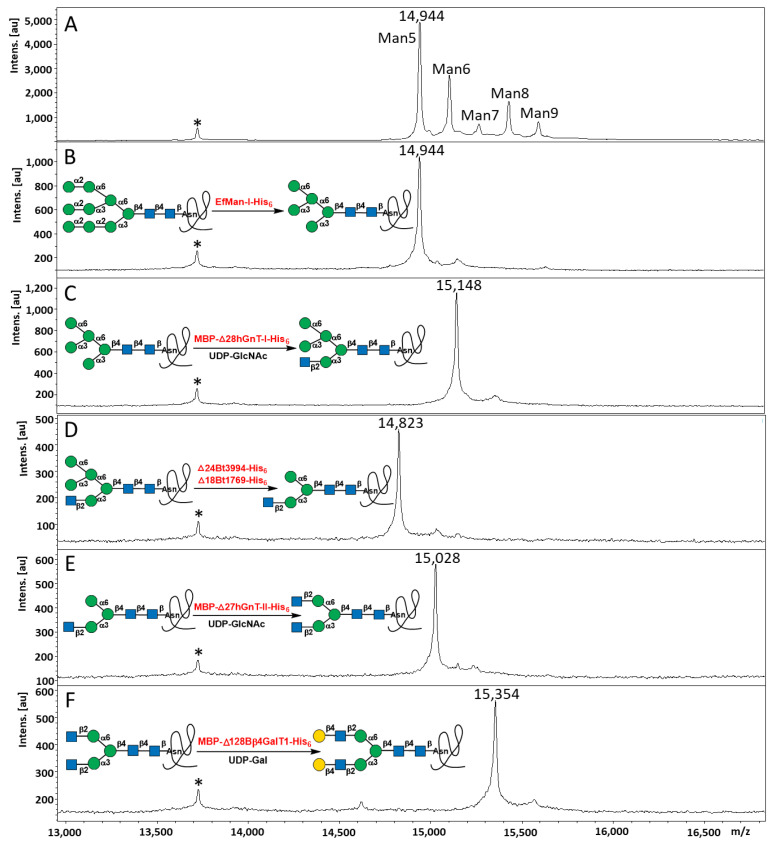
MALDI-TOF MS analysis results for RNase B with heterogeneous high-mannose N-glycans (**A**) and RNase B sequentially treated with EfMan-I-His_6_ (**B**), MBP-∆28hGnT-I-His_6_ (**C**), ∆24Bt3994-His_6_ and ∆18Bt1769-His_6_ (**D**), MBP-∆27hGnT-II-His_6_ (**E**), and MBP-∆128Bβ4GalT1-His_6_ (**F**) in step-wise reactions. The peak marked with an asterisk (*) in each figure is for RNase A, the non-glycosylated ribonuclease that is present in the commercially obtained RNase B sample, which was used as the internal standard. The schematic illustrations of the corresponding reactions are shown. The number above each peak represents the *m*/*z* value of the expected product + 2Na species.

As shown in Figure 3, treating RNase B (5 mg/mL) (Figure 3A) with 3% (*w*/*w*) EfMan-I-His_6_ at 30 °C for 2 h, the high-mannose-type N-glycans Man_5–9_GlcNAc_2_ on RNase B were completely trimmed down to Man_5_GlcNAc_2_ (Figure 3B). Treating the resulting RNase B sample with 3% (*w*/*w*) MBP-∆28hGnT-I in the presence of MnCl_2_ (2 mM) and UDP-GlcNAc (1 mM) at 30 °C for 2 h completed the reaction for the formation of RNase B with a homogeneous GlcNAc_1_Man_5_GlcNAc_2_ N-glycan (Figure 3C). Incubation of the resulting RNase B with 4% (*w*/*w*) ∆24Bt3994-His_6_ and 3% (*w*/*w*) ∆18Bt1769-His_6_ in the presence of 2 mM CaCl_2_ at 30 °C for 2 h completed the cleavage of the terminal α1–6- and α1–3-linked mannose residues to form RNase B with a homogeneous GlcNAc_1_Man_3_GlcNAc_2_ N-glycan, which was not cleaved further in the presence of both ∆24Bt3994-His_6_ and ∆18Bt1769-His_6_ (Figure 3D). The resulting reaction mixture was incubated with MBP-∆27hGnT-II-His_6_ (10% *w*/*w*) in the presence of 2 mM MnCl_2_ and 1 mM UDP-GlcNAc at 30 °C overnight to form RNase B containing homogeneous GlcNAc_2_Man_3_GlcNAc_2_ N-glycan (Figure 3E). MBP-∆128Bβ4GalT1-His_6_ (3% *w*/*w*) was then used to process the N-glycan in the resulting RNase B to form RNase B containing homogeneous Gal_2_GlcNAc_2_Man_3_GlcNAc_2_ N-glycan by incubating at 30 °C in 2 h in the presence of 5 mM MnCl_2_ and 2 mM UDP-Gal (Figure 3F). Notably, all enzymes used for RNase B N-glycan processing were active in the presence of Tris-HCl (100 mM, pH 7.5).

### 2.4. Multi-Step OPME N-Glycan Processing

Due to the high specificity of the acceptor substrate preference of mammalian glycosyltransferases and the high efficiency of the bacterial mannosidases used in the glycoprotein in vitro N-glycan processing described above, we hypothesized that the step-by-step process was not necessary, and one-pot approaches were possible and could be more efficient. To test this hypothesis, a series of one-pot multienzyme (OPME) reactions were carried out and the resulting RNase B samples were analyzed directly by MALDI-TOF MS assays after dialysis.

**Figure 4:**
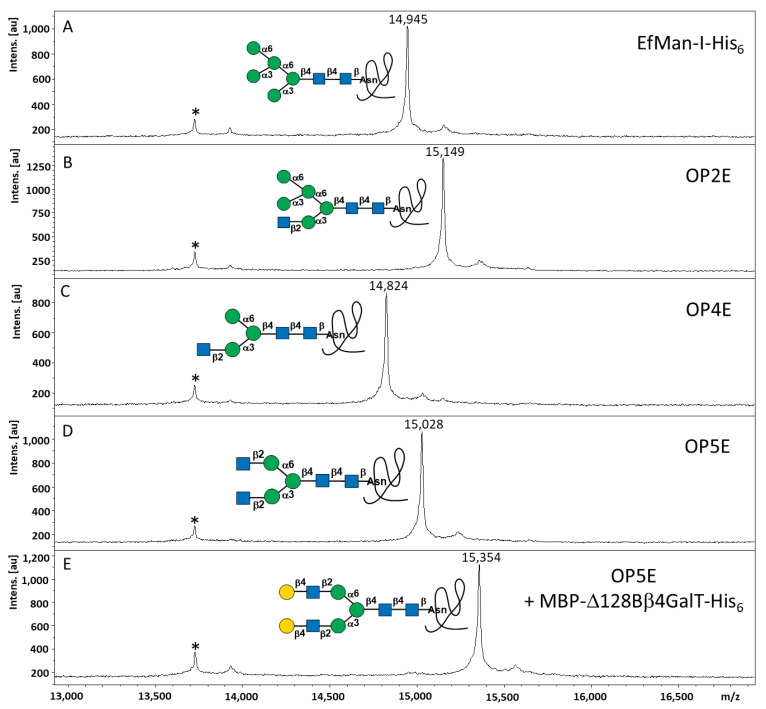
MALDI-TOF MS analysis results for RNase B treated with EfMan-I-His_6_ for 2 h (**A**), OP2E containing EfMan-I-His_6_, MBP-∆28hGnT-I-His_6_ for 2 h (**B**), OP4E containing EfMan-I-His_6_, MBP-∆28hGnT-I-His_6_, ∆24Bt3994-His_6_, and ∆18Bt1769-His_6_ for 2 h (**C**), OP5E containing EfMan-I-His_6_, MBP-∆28hGnT-I-His_6_, ∆24Bt3994-His_6_, ∆18Bt1769-His_6_, and MBP-∆27hGnT-II-His_6_ for 18 h (**D**), and OP5E for 18 h followed by incubation with MBP-∆128Bβ4GalT1-His_6_ for additional 2 h (**E**). All reactions were carried out at 30 °C in Tris-HCl (100 mM, pH 7.5) containing CaCl_2_ (2 mM), MgCl_2_ (2 mM), and MnCl_2_ (2 mM). The peak marked with an asterisk (*) in each figure is for RNase A, the non-glycosylated ribonuclease that is present in the commercially obtained RNase B sample, which was used as the internal standard. The symbol representations of the expected products are shown.

Indeed, reactions with EfMan-I-His_6_ (3% *w*/*w*) alone, one-pot two-enzyme (OP2E) reactions containing EfMan-I-His_6_ (3% *w*/*w*) and MBP-∆28hGnT-I-His_6_ (3% *w*/*w*), as well as one-pot four-enzyme (OP4E) containing EfMan-I-His_6_ (3% *w*/*w*), MBP-∆28hGnT-I-His_6_ (3% *w*/*w*), ∆18Bt1769-His_6_ (3% *w*/*w*), and ∆24Bt3994-His_6_ (4% *w*/*w*) were very efficient. The RNase B products containing the expected N-glycan structures were all obtained in 2 h at 30 °C (Figure 4A–C). In comparison, one-pot five-enzyme (OP5E) reactions containing EfMan-I-His_6_ (3% *w*/*w*), MBP-∆28hGnT-I-His_6_ (3% *w*/*w*), ∆18Bt1769-His_6_ (3% *w*/*w*), ∆24Bt3994-His_6_ (4% *w*/*w*), and MBP-∆27hGnT-II-His_6_ (10% *w*/*w*) were slower, and the RNase B product containing the target N-glycan was obtained after incubation at 30 °C overnight (~18 h) (Figure 4D). This indicated that the addition of the second GlcNAc catalyzed by MBP-∆27hGnT-II-His_6_ was the rate limiting step of the process in this system. It is worth mentioning that further treatment of the OP4E product with ∆24Bt3994-His_6_ (5% *w*/*w*) or ∆18Bt1769-His_6_ (3% *w*/*w*) at 30 °C overnight did not lead to further cleavage of the N-glycan (data not shown), highlighting the applicable combination of ∆18Bt1769-His_6_ and ∆24Bt3994-His_6_ in glycoprotein N-glycan processing from high-mannose N-glycans without the concern of removing extra mannose residues.

**Figure 5:**
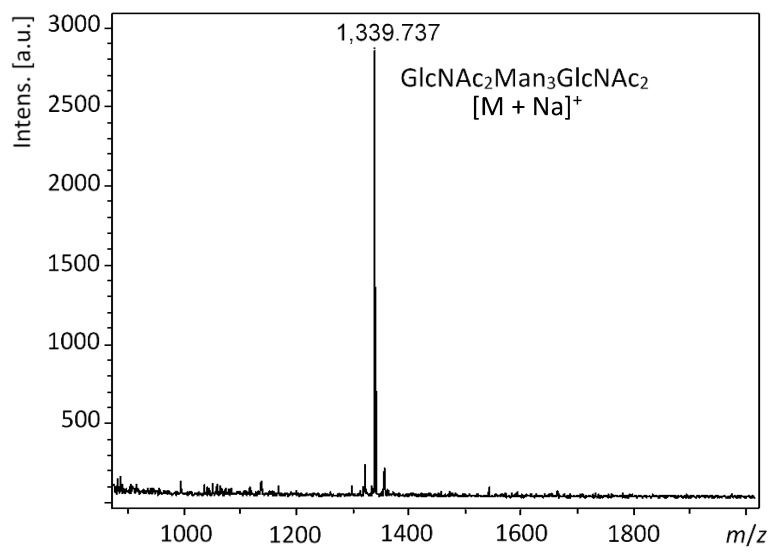
MALDI-TOF MS analysis result of the N-glycan released from the RNase B product of OP5E reactions; *m*/*z* calculated for the sodium adduct of the product GlcNAc_2_Man_3_GlcNAc_2_ [M + Na]^+^ was 1339.476, found 1339.737.

The N-glycan of the RNase B product of the OP5E reaction was released by peptide:N-glycosidase F (PNGase F) and analyzed by MALDI-TOF MS assays. The results (Figure 5) were consistent with those obtained with the intact RNase B samples. Only the target N-glycan GlcNAc_2_Man_3_GlcNAc_2_ (+Na, *m*/*z* = 1339.737) was observed without the presence of other N-glycans, confirming the efficiency of the OP5E reactions and the N-glycan homogeneity of the RNase B glycoprotein obtained.

Incubating the reaction mixture of the OP5E reaction with UDP-Gal (2 mM) and MBP-∆128Bβ4GalT1-His_6_ (3% *w*/*w*) at 30 °C for 2 h completed the formation of RNase B with Gal_2_GlcNAc_2_Man_3_GlcNAc_2_ N-glycan (Figure 4E).

**Figure 6:**
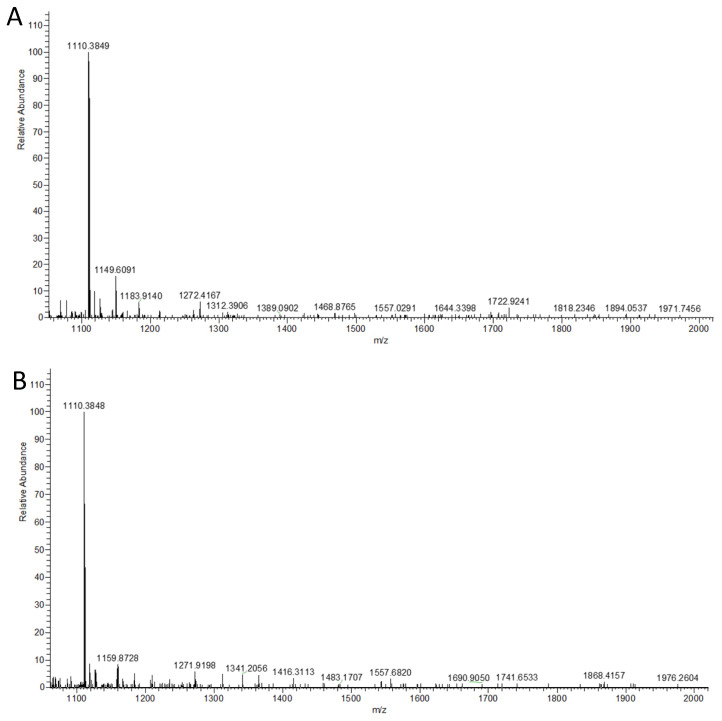
HRMS (negative mode) assay results for the N-glycans released from the RNase B products produced by a one-pot two-enzyme (OP2E) sialylation reaction containing *Neisseria meningitidis* CMP-sialic acid synthetase (NmCSS) and MBP-∆89ST6GAL-I-His_6_ (**A**) or MBP-CjCst-I∆145-His_6_ (**B**). The *m*/*z* value expected for the ionized disialyl N-glycan (Neu5Ac_2_Gal_2_GlcNAc_2_Man_3_GlcNAc_2_) was 1110.3842. The ionized substrate (*m*/*z* value expected 1663.5819), substrate + Cl (*m*/*z* value expected 1675.5610), or monosialylated N-glycan (*m*/*z* value expected 1930.6803) was not observed.

The formation of RNase B with disialylated biantennary complex N-glycan was accomplished by incubating with MBP-∆89hST6GAL-I-His_6_ (3% *w*/*w*) or MBP-CjCst-I∆145-His_6_ (3% *w*/*w*) in the presence of cytidine 5′-monophosphate-Neu5Ac (CMP-Neu5Ac, 5 mM) for the formation of α2–6 or α2–3-sialyl linkage, respectively. It was observed that sialic acids were cleaved from sialosides during MALDI-TOF analysis. Therefore, high-resolution mass spectrometry (HRMS) analysis was used to analyze the N-glycans released from RNase B sialylation products by PNGase F digestion and purified by Cotton HILIC SPE microtips [60]. We found that in situ generation of CMP-Neu5Ac from Neu5Ac and CTP by *Neisseria meningitides* CMP-sialic acid synthetase (NmCSS) [61] during enzymatic sialylation process in a one-pot two-enzyme system [62] further improved the efficiency of sialylation. As shown in Figure 6, the ionized species of disialyl biantennary N-glycans released from RNase B sialyation products (Neu5Ac_2_Gal_2_GlcNAc_2_Man_3_GlcNAc_2_, *m*/*z* value expected 1110.3842; *m*/*z* values observed were 1110.3849 and 1110.3848 for the double-charged α2–6 and α2–3-linked species, respectively) were clearly observed. Ionized species for monosialylated N-glycan (Neu5Ac_1_Gal_2_GlcNAc_2_Man_3_GlcNAc_2_, *m*/*z* value expected: 1930.6803) and N-glycan released from the RNase B substrate for sialylation reactions (Gal_2_GlcNAc_2_Man_3_GlcNAc_2_, *m*/*z* value expected 1663.5819, or 1675.5610 for its Cl adduct) were not observed. This indicated that the di-sialylation reactions went to completion.

## 3. Conclusions

Using RNase B as a model glycoprotein substrate, we have successfully established a glycoprotein in vitro N-glycan processing platform for the production of glycoproteins containing homogeneous α2–6- or α2–3-linked disialylated biantennary complex N-glycans using enzymes expressed in *E. coli*. Several mammalian glycoprotein N-glycan processing glycosyltransferases including hGnT-I, hGnT-II, Bβ4GalT1, and hST6GAL-I have been successfully expressed in *E. coli* Origami B (DE3) cells as N-terminal MBP-fused and C-terminal His_6_-tagged fusion proteins in a soluble and active form. In addition, bacterial mannosidases including EfManI, Bt3994, and Bt1769, as well as a bacterial sialyltransferase CjCst-I have been successfully expressed as His_6_-tagged proteins. These enzymes can be easily purified using a single Ni^2+^-column. The in vitro processing of the high-mannose-type N-glycans in glycoprotein RNase B has been successfully achieved with the combination of these enzymes used in sequential step-wise reactions or in one-pot reactions. The platform developed can find broad applications for producing glycoproteins with homogeneous N-glycoforms.

## 4. Materials and Methods

### 4.1. Materials and Instruments

Chemicals were purchased and used as received without further purification. Matrix-assisted laser desorption/ionization mass spectra were obtained using Bruker UltraFlextreme MALDI-TOF/TOF (Bruker, Billerica, MA, USA) and high-resolution mass spectrometry results were obtained using Thermo Q-Exactive HF (Thermo Fisher Scientific, Asheville, NC, USA) at the University of California-Davis Campus Mass Spectrometry Facilities. Proteins were purified using NGC medium-pressure liquid chromatography systems (Bio-Rad, Hercules, CA, USA). Peptide N-glycosidase F (PNGase F) was obtained from R&D Systems, Minneapolis, Minnesota, USA. Vector plasmids pMAL-c4X and pMAL-c2X were from New England Biolabs, Inc., Ipswich, MA, USA Vector plasmids pET15b and pET22b(+) were from Novagen, Madison, WI, USA. Profinity and Nuvia IMAC Ni-charged resins, and mini Nuvia IMAC cartridges (5 mL) were from Bio-Rad, Hercules, California, USA. Galactose, *N*-acetylglucosamine (GlcNAc), LB media, isopropyl-1-thio-D-galactopyranoside (IPTG), and ampicillin were from Fisher Scientific, Inc., Hampton, NH, USA. Kanamycin sulfate was from AMRESCO, Fairlawn, NJ, USA. Chloramphenicol was from ALDRICH, St. Louis, MO, USA. Tetracycline hydrochloride was from Calbiochem, La Jolla, CA, USA. *N*-Acetylneuraminic acid (Neu5Ac) was from Inalco (Milano, Italy). Adenosine 5′-triphosphate (ATP), cytosine 5′-triphosphate (CTP), and uridine 5′-triphosphate (UTP) were purchased from Hangzhou Meiya Pharmaceutical Co. Ltd. (Hangzhou, China). DH5α competent cells, BL21 (DE3) competent cells, restriction enzymes, GeneJET Gel Extraction Kit, GeneJET Plasmid Miniprep Kit, Phusion™ Plus DNA Polymerase, T4 ligase, and 2,5-dihydroxybenzoic acid (2,5-DHB) were purchased from Thermo Fisher Scientific, Asheville, NC, USA. Sinapinic (or sinapic) acid was from TCI America, Portland, OR, USA. Origami B (DE3) competent cells were from Novagen and pGro7 was from Takara Bio USA Inc, Mountain View, CA, USA. Herculase-enhanced DNA polymerase was from Agilent Technologies, Santa Clara, California, USA. UDP-GlcNAc [63], UDP-Gal [64], and CMP-Neu5Ac [61] were prepared as described previously.

### 4.2. Cloning

All polymerase chain reactions (PCRs) were carried out with Phusion^®^ HF DNA polymerase by following the standard protocol provided by the manufactory unless noted. Briefly, the reaction was performed in a reaction mixture (50 μL) containing a template (10 ng plasmid or synthetic DNA, or 1 μg of genomic DNA), 10 × Phusion^®^ HF buffer (5 μL), dNTP mixture (1 mM each), and 5 U (1 μL) of Phusion^®^ HF DNA polymerase, forward and reverse primers (1 μM each). The reaction mixture was subjected to 30 cycles of amplification. The primers and the annealing temperature (Ta) used for each PCR reaction are listed in Table 2. The PCR products were purified by GeneJET Gel Extraction Kit and digested with two restriction enzymes at 37 °C for 2 h. The digested products were purified by GeneJET Gel Extraction Kit and ligated with vector plasmid pre-digested with the same restriction enzymes and similarly gel extraction purified. The ligation was carried out at 16 °C overnight using T4 DNA ligase. The ligated product was transformed into the chemical competent *E. coli* DH5α cells. Plasmids were purified using GeneJET Plasmid Miniprep Kit and sequences were confirmed by DNA sequencing (See Supplementary Materials). Positive plasmids were selected and transferred to chemically competent *E. coli* BL21 (DE3) or Origami B (DE3) cells for expression.

MBP-∆28hGnT-I-His_6_: A synthetic gene encoding an N-terminal 28 amino acid truncated human GnT-I (hGnT-I, GenBank accession number: NP_001108089.1) (∆28hGnT-I) with codon optimized for *E. coli* expression was cloned in pET15b vector to construct plasmid pET15b-∆28hGnT-I. PCR was performed with 5 U (1 μL) of Herculase-enhanced DNA polymerase in Herculase buffer and other conditions were the same as described above. The resulting plasmid was used as the PCR template to construct pMAL-c2X-∆28hGnT-I in pMAL-c2X vector for expressing MBP-∆28hGnT-I-His_6_.

∆18Bt1769-His_6_ and ∆24Bt3994-His_6_: Full-length Bt3994 and Bt1769 [40] from *B. thetaiotaomicron* genomic DNA (ATCC 2914D-5) were cloned into the pET22b (+) vector to construct pET22b (+)-Bt3994 and pET22b (+)-Bt1769, respectively. The resulting plasmids were used as PCR templates to construct pET22b (+)-∆18Bt1769 and pET22b (+)-∆24Bt3994, respectively, for the expression of C-His_6_-tagged truncated mannosidases ∆24Bt3994 (residues 25–743) and ∆18Bt1769 (residues 19–751).

MBP-∆27hGnT-II-His_6_: A synthetic gene encoding an N-terminal∆27 amino acid truncated human GnT-II (hGnT-II, GenBank accession number: NP_002399.1) was cloned in pMAL-c4X vector to construct pMAL-c4X-∆27hGnT-II for expressing MBP-∆27hGnT-II-His_6_.

MBP-∆128Bβ4GalT1-His_6_: Previously obtained plasmid pET15b-∆128Bβ4GalT1 [35] was used as a PCR template to construct pMAL-c4X-∆128Bβ4GalT1 in pMAL-c4X vector for expressing N-terminal 128 amino acid truncated bovine β1–4GalT1 (GenBank accession number: XP_019821962.1) as an N-terminal MBP-fused and C-terminal His_6_-tagged fusion protein MBP-∆128Bβ4GalT1-His_6_.

MBP-∆89hST6GAL-I-His_6_: A synthetic gene of an N-terminal l89 amino acid truncated human ST6GAL-I (hST6GAL-I, GenBank accession number: NP_001340845.1) with codon optimized for *E. coli* expression was cloned in pMAL-c2X vector to construct plasmid pMAL-c2X-∆89hST6GAL-I for expressing MBP-∆89hST6GAL-I-His_6_.

MBP-CjCst-I∆145-His_6_: A synthetic gene of C-terminal 145 amino acids truncated *Campylobacter jejuni* Cst-I (CjCst-I, GenBank accession number: AAF13495.1) with codon optimized for *E. coli* expression was cloned into pMAL-c4X to construct plasmid pMAL-c4X-CjCst-I∆145 for expressing MBP-CjCst-I∆145-His_6_.

### 4.3. Enzyme Expression and Purification

To express enzymes in *E. coli* BL21 (DE3) expression system, cells harboring the plasmid of interest were cultured in LB media (10 g/L tryptone, 5 g/L yeast extract, and 10 g/L NaCl) supplemented with ampicillin (100 µg/mL). When the OD_600 nm_ of the culture reached 0.6–0.8, isopropyl-1-thio-D-galactopyranoside (IPTG, 0.1 mM) was added and the culture was incubated at 20 °C for 20 h. To express enzymes in *E. coli* Origami B (DE3) harboring pGro7 and the plasmid of interest, cells were cultured in LB media supplemented with ampicillin (50 mg/mL), tetracycline (5 mg/L), chloramphenicol (17 mg/L), kanamycin (25 mg/L), and L-arabinose (1 g/L, for chaperon expression). When the OD_600 nm_ of the culture reached 0.6–0.8, IPTG (0.1 mM) was added and the culture was incubated at 16 °C for 48 h.

After the expression was completed, cells were harvested by centrifugation (6000× *g*) at 4 °C for 30 min and re-suspended in lysis buffer (50 mM Tris-HCl, pH 8.0, 300 mM NaCl, 0.1% Triton X-100). The cell resuspension was subjected to the homogenizer (EmulsiFlex-C3) to break the cells. The cell lysate was obtained as the supernatant after centrifugation (9016× *g*) at 4 °C for 60 min and purified by a Ni^2+^-NTA affinity column, such as a mini Nuvia IMAC cartridge (5 mL), on a Bio-Rad NGC system. The column was pre-equilibrated with 6 column volumes of binding buffer containing Tris-HCl buffer (50 mM, pH 8.0), NaCl (300 mM). It was washed with 10 column volumes of binding buffer, followed by washing with 10 column volumes of 10% elute buffer, and 10 column volumes of 20% elute buffer, and then eluted with elute buffer containing Tris-HCl (50 mM, pH 8.0), NaCl (300 mM), imidazole (250 mM). The fractions containing the purified protein were combined for dialysis against a dialysis buffer (Tris-HCl, 50 mM, pH 7.5, 250 mM NaCl) or for concentration using a protein concentrator (10 KDa cut-off). Finally, 20% glycerol (for MBP-∆128Bβ4GalT1-His_6_) or 10% glycerol (for other enzymes) was added before storing the samples at −20 °C.

### 4.4. Stepwise Enzymatic Reaction Using RNase B as The Substrate

RNase B (5 mg/mL, 330 μM) was incubated with EfMan-I-His_6_ (150 μg/mL, 1.8 μM) in Tris-HCl (100 mM, pH 7.5) at 30 °C for 2 h. The resulting mixture was then dialyzed against ddH_2_O and subjected to MALDI-TOF MS analysis. It was also used for the assays described below.

The dialyzed EfMal-I-His_6_-treated RNase B (~330 μM) was used as the acceptor substrate. It was incubated with UDP-GlcNAc (1 mM) and MBP-∆27hGnT-I-His_6_ (1.6 μM) in Tris-HCl (100 mM, pH 7.5) containing MnCl_2_ (2 mM) at 30 °C for 2 h. The weight ratio of MBP-∆27hGnT-I-His_6_ to RNase B was 3% (*w*/*w*). The resulting mixture was then dialyzed against ddH_2_O and subjected to MALDI-TOF MS analysis. It was also used for the assays described below.

The RNase B obtained above was incubated with ∆24Bt3994-His_6_ (1.9 μM) and ∆18Bt1769-His_6_ (1.8 μM) in Tris-HCl (100 mM, pH 7.5) containing CaCl_2_ (2 mM) at 30 °C for 2 h. The weight ratios of ∆24Bt3994-His_6_ and ∆18Bt1769-His_6_ to RNase B were 4% (*w*/*w*) and 3% (*w*/*w*), respectively. The resulting mixture was then dialyzed against ddH_2_O and subjected to MALDI-TOF MS analysis. It was also used for the assays described below.

The RNase B obtained above was incubated with UDP-GlcNAc (2 mM) and MBP-∆27hGnT-II-His_6_ (3.2 μM) in Tris-HCl (100 mM, pH 7.5) containing MnCl_2_ (2 mM) at 30 °C for 2 h. The weight ratio of ∆27hGnT-II to RNase B was 10% (*w*/*w*). The resulting mixture was then dialyzed against ddH_2_O and subjected to MALDI-TOF MS analysis. It was also used for the assays described below.

The RNase B obtained above was incubated with UDP-Gal (2 mM) and MBP-∆128Bβ4GalT1-His_6_ (3.9 μM) in Tris-HCl (100 mM, pH 7.5) containing MnCl_2_ (2 mM) at 30 °C for 2 h. The weight ratio of MBP-∆128Bβ4GalT1-His_6_ to RNase B was 10% *w*/*w*. The resulting mixture was then dialyzed against ddH_2_O and subjected to MALDI-TOF MS analysis.

Dialysis was not necessary for step-wise reactions, but was preferred before MALDI-TOF MS analysis of the samples.

### 4.5. OPME Reactions for N-Glycan Processing of RNase B

One-pot two-enzyme (OP2E) reactions were carried out in a 0.5 mL microcentrifuge tube by incubating RNase B (330 μM), UDP-GlcNAc (2 mM), EfMan-I-His_6_ (1.8 μM, 3% *w*/*w*), and MBP-∆28hGnT-I-His_6_ (1.6 μM, 3% *w*/*w*), in Tris-HCl (100 mM, pH 7.5) containing MnCl_2_ (2 mM), CaCl_2_ (2 mM), MnCl_2_ (2 mM) at 30 °C for 2 h.

One-pot two-enzyme (OP2E) reactions were carried out similarly as described above for OP2E reactions, except for the fact that two more enzymes including ∆24Bt3994-His_6_ (1.9 μM, 4% *w*/*w*) and ∆18Bt1769-His_6_ (1.8 μM, 3% *w*/*w*) were added.

One-pot two-enzyme (OP5E) reactions were carried out similarly to OP4E reactions except for the fact that one more enzyme MBP-∆27hGnT-II-His_6_ (3.2 μM, 10% *w*/*w*) was added and the reaction was continued overnight.

Galactosylation was accomplished by adding UDP-Gal (2 mM) and MBP-∆128Bβ4GalT1-His_6_ (3.9 μM, 10% *w*/*w*) to the reaction mixture after OP5E reaction followed by incubation at 30 °C for 2 h.

The final step α2–6-sialylation was completed by adding CMP-Neu5Ac (5 mM) and MBP-∆89hST6GAL-I-His_6_ (1.9 μM, 3% *w*/*w*) to the reaction mixture after galactosylation followed by incubation at 30 °C overnight.

Similarly, α2–3-sialylation was completed by adding CMP-Neu5Ac (5 mM) and MBP-CjCst-I∆145-His_6_ (1.9 μM, 3% *w*/*w*) to the reaction mixture after galactosylation followed by incubation at 30 °C for a shorter time of 2 h.

For sialylation reactions with in situ generation of the sialyltransferase donor, CMP-Neu5Ac (5 mM) was replaced by Neu5Ac (5 mM), CTP (7.5 mM), and NmCSS (5 μM). Other conditions were the same as described above.

### 4.6. MALDI-TOF MS Analyses of RNase B samples and the Released N-Glycans

Fresh solutions of 2,5-DHB (15 mg/mL) dissolved in ddH_2_O and sinapinic acid (SA) (20 mg/mL) dissolved in ACN/0.1%TFA (7:3) were prepared. A mixed solution of these two with a 1:1 (*v*/*v*) ratio was used as the matrix for MALDI-TOF MS analysis of RNase B samples (~1 mg/mL) dialyzed against ddH_2_O using Slide-A-Lyzer™ MINI Dialysis (10 k MWCO) devices.

To release N-glycans from RNase B samples, RNase B (1 mg) was denatured by adding 0.5% SDS and dithiothreitol (DTT) (40 mM) in 200 μL followed by incubation at 98 °C for 10 min and then at room temperature for 5 min. PNGase F (100 ng) was then added and the glycans were released by incubation at 37 °C for 2 h. The deglycosylated proteins were precipitated by adding three volumes of pre-chilled ethanol followed by incubation on ice for 20 min. The mixtures were then centrifuged at 16,200× *g* for 5 min and the supernatants containing the glycans were purified with graphitized carbon cartridge and dried in a speed vacuum. They were dissolved in ddH_2_O and used for MALDI-TOF MS analysis using 2,5-DHB dissolved in ACN/0.1%TFA (7:3) (25 mg/mL) as the matrix.

### 4.7. HRMS Analysis of Sialylated N-Glycans

RNase B (100 μg) samples were denatured and the N-glycans were released by treating with PNGase F (10 ng) similar to the conditions described above. The samples were cleaned using a homemade cotton tip via hydrophilic interaction liquid chromatography-solid phase extraction (HILIC-SPE) [60]. Briefly, samples were mixed with acetonitrile (ACN, 50% *v*/*v*) centrifuged (16,200× *g*) at 4 °C for 5 min. The supernatant was transferred to a clean tube, pipetting up-and-down for a total of 20 times in a 10 μL-tip packed with a small volume of cotton. The cotton tip was washed 3 times with 20 μL of 85% ACN with 1% TFA, followed by 3 times with 20 μL of 85% ACN, and eluted with ddH_2_O (10 μL). The eluant was used for HRMS analysis.

## Figures and Tables

**Table 1:** Enzymes obtained for glycoprotein in vitro N-glycan processing and their expression levels in *E. coli* BL21 (DE3) or Origami B (DE3) cells.

Enzyme	CAZy Family	Source	Expression Host	Expression Level (mg/L LB)
EfMan-I-His_6_	GH92	*Enterococcus faecalis V583*	BL21 (DE3)	85 ^[a]^
∆24Bt3994-His_6_	GH92	*Bacteroides thetaiotaomicron*	BL21 (DE3)	55
∆18Bt1769-His_6_	GH92	*Bacteroides thetaiotaomicron*	BL21 (DE3)	55
MBP-∆28hGnT-I-His_6_	GT13	Human	Origami B (DE3)/pGro7	5
MBP-∆27hGnT-II-His_6_	GT16	Human	Origami B (DE3)/pGro7	~1
MBP-∆128Bβ4GalT1-His_6_	GT7	Bovine	Origami B (DE3)/pGro7	60
MBP-∆89ST6GAL-I-His_6_	GT29	Human	Origami B (DE3)/pGro7	30
MBP-CjCst-I∆145-His_6_	GT42	*Campylobacter jejuni*	BL21 (DE3)	60

^[a]^ As reported in Ref. [34].

**Table 2:** Primers used for cloning. Restriction sites are italicized.

Plasmid	Ta (°C)	Primer Sequences (5′to 3′)		Restriction Enzyme
pET15b-∆28hGnT-I	52	Forward	ATC*CATATG*TGGACCCGTCCAGCCCCAGGT	NdeⅠ
		Reverse	CCG*GGATCC*TTAATTCCAGCTTGGATCATAACCTTC	BamHI
pMAL-c2X-∆28hGnT-I	52	Forward	GACC*GAATTC*TGGACCCGTCCAGCCCCAGGT	EcoRI
		Reverse	CAGC*AAGCTT*TTAGTGGTGGTGATGATGATGATTCCAGCTTGGATCATAACCTTC	HindIII
pET22b(+)-Bt3994	55	Forward	CTCCAG*CATATG*CTCCAGCATATGAAAACACCTATTTACCT	NdeI
		Reverse	CTCCAG*CTCGAG*CTGGGTCTGTTCTTCAAATTCG	XhoI
pET22b(+)-∆24Bt3994	55	Forward	CTCCAG*CATATG*CTCCAGCATATGAAAACACCTATTTACCT	NdeI
		Reverse	CTCCAG*CTCGAG*CTGGGTCTGTTCTTCAAATTCG	XhoI
pET22b(+)-Bt1769	55	Forward	CTCCAG*CATATG*AAACTTACACACATTTT	NdeI
		Reverse	CTCCAG*CTCGAG*CTTGATTTCTAATGATGGAGG	XhoI
pET22b(+)-∆18Bt1769	55	Forward	CTCCAG*CATATG*AAACTTACACACATTTT	NdeI
		Reverse	CTCCAG*CTCGAG*CTTGATTTCTAATGATGGAGG	XhoI
pMAL-c4x-∆27hGnT-II	56	Forward	GATC*GGATCC*AACGGTCGTCAGCGTAAAAA	BamHI
		Reverse	CGG*AAGCTT*TTAGTGGTGGTGGTGGTGGTGCTGCAGGCGGCGATAGG	HindIII
pMAL-c4X-∆128Bβ4GalT1	55	Forward	GACC*GAATTC*CGCTCGCTGACCGCATG	EcoRI
		Reverse	CAGC*AAGCTT*TCAATGGTGATGGTGATGGTGGCTCGGCGTCCCGATGTC	HindIII
pMAL-c2X-∆89hST6GAL-I	50	Forward	GACC*GAATTC*GAGGCCTCGTTTCAGGTTTG	EcoRI
		Reverse	CAGC*AAGCTT*TTAGTGGTGGTGATGATGATGGCAGTGTATTGTTCGGAATC	HindIII
pMAL-c4x-CjCst-I∆145	56	Forward	CTCTCT*GAATTC*ATGACACGCACCAGAATGGAG	EcoRI
		Reverse	CTCTCT*GTCGAC*TTAATGATGATGATGATGATGATAAAAGTTCAGCATTAT	SalI

## Data Availability

Not applicable.

## Supplementary Materials

The following supporting information can be downloaded at: https://www.mdpi.com/article/10.3390/molecules28062753/s1. The DNA and amino acid sequences of MBP-∆28hGnT-I-His_6_, ∆24Bt3994-His_6_, ∆18Bt1769-His_6_, MBP-∆27hGnT-II-His_6_, MBP-∆128Bβ4GalT1-His_6_, MBP-∆89hST6GAL-I-His_6_, and MBP-CjCst-I∆145-His_6_.
