# Supplementary material for: Glycoprotein In Vitro N-Glycan Processing Using Enzymes Expressed in E. coli
Source: Molecules. 2023 Mar 18;28(6):2753. doi: 10.3390/molecules28062753 (PMC10051842; doi:10.3390/molecules28062753)
Supplement: Supplementary file 1 [file molecules-28-02753-s001.zip › molecules-2226396-supplementary.pdf]

# Supporting Information

## Glycoprotein *in vitro* N-Glycan Processing Using Enzymes Expressed in *E. coli*

Libo Zhang [1†], Yanhong Li [1†,] Riyao Li [1†], Xiaohong Yang [1], Zimin Zheng [1], Jingxin Fu [1], Hai Yu [1], and Xi Chen [1,*]

[1]Department of Chemistry, University of California, Davis, California, United States
*Correspondence: xiichen@ucdavis.edu; Tel.: 1-530-754-6037

**Table of Contents**

**Figure S1.** The DNA and the amino acid sequences of MBP-Δ28hGnT-I-$His_6$…………………...**S2**

**Figure S2.** The DNA and the amino acid sequences of Δ24Bt3994-$His_6$…………………………**S3**

**Figure S3.** The DNA and the amino acid sequences of Δ18Bt1769-$His_6$ …………………………**S4**

**Figure S4.** The DNA and the amino acid sequences of MBP-Δ27hGnT-II-$His_6$…………………**S5**

**Figure S5.** The DNA and the amino acid sequences of MBP-Δ128Bβ4GalT1-$His_6$………………**S6**

**Figure S6.** The DNA and the amino acid sequences of MBP-Δ89hST6GAL-I-$His_6$……………...**S7**

**Figure S7.** The DNA and the amino acid sequences of MBP-CjCst-IΔ145-$His_6$…………………**S8**

**Figure S1.** The DNA (**a**) and the amino acid (**b**) sequences of MBP-Δ28hGnT-I-His$_6$. The sequences from the vector are underlined. The linker and the His$_6$-tag sequences are shown in bold.

**(a)**

ATGAAAATCGAAGAAGGTAAACTGGTAATCTGGATTAACGGCGATAAAGGCTATAACGGTCTCGCT
GAAGTCGGTAAGAAATTCGAGAAAGATACCGGAATTAAAGTCACCGTTGAGCATCCGGATAAACTG
GAAGAGAAATTCCCACAGGTTGCGGCAACTGGCGATGGCCCTGACATTATCTTCTGGGCACACGAC
CGCTTTGGTGGCTACGCTCAATCTGGCCTGTTGGCTGAAATCACCCCGGACAAAGCGTTCCAGGAC
AAGCTGTATCCGTTTACCTGGGATGCCGTACGTTACAACGGCAAGCTGATTGCTTACCCGATCGCT
GTTGAAGCGTTATCGCTGATTTATAACAAAGATCTGCTGCCGAACCCGCCAAAAACCTGGGAAGAG
ATCCCGGCGCTGGATAAAGAACTGAAAGCGAAAGGTAAGAGCGCGCTGATGTTCAACCTGCAAGAA
CCGTACTTCACCTGGCCGCTGATTGCTGCTGACGGGGGTTATGCGTTCAAGTATGAAAACGGCAAG
TACGACATTAAAGACGTGGGCGTGGATAACGCTGGCGCGAAAGCGGGTCTGACCTTCCTGGTTGAC
CTGATTAAAAACAAACACATGAATGCAGACACCGATTACTCCATCGCAGAAGCTGCCTTTAATAAA
GGCGAAACAGCGATGACCATCAACGGCCCGTGGGCATGGTCCAACATCGACACCAGCAAAGTGAAT
TATGGTGTAACGGTACTGCCGACCTTCAAGGGTCAACCATCCAAACCGTTCGTTGGCGTGCTGAGC
GCAGGTATTAACGCCGCCAGTCCGAACAAAGAGCTGGCAAAAGAGTTCCTCGAAAACTATCTGCTG
ACTGATGAAGGTCTGGAAGCGGTTAATAAAGACAAACCGCTGGGTGCCGTAGCGCTGAAGTCTTAC
GAGGAAGAGTTGGCGAAAGATCCACGTATTGCCGCCACTATGGAAAACGCCCAGAAAGGTGAAATC
ATGCCGAACATCCCGCAGATGTCCGCTTTCTGGTATGCCGTGCGTACTGCGGTGATCAACGCCGCC
AGCGGTCGTCAGACTGTCGATGAAGCCCTGAAAGACGCGCAGACT**AATTCGAGCTCGAACAACAAC**
**AACAATAACAATAACAACAACCTCGGGATCGAGGGAAGGATTTCAGAATTC**TGGACCCGTCCAGCC
CCAGGTCGCCCACCAAGCGTTAGCGCCCTGGATGGCGACCCAGCAAGCCTGACCCGTGAAGTGATT
CGTCTGGCGCAGGACGCCGAAGTTGAACTGGAGCGTCAACGCGGTCTGCTGCAACAAATCGGCGAC
GCGCTGAGCAGCCAGCGTGGCCGTGTTCCAACGGCAGCACCACCGGCCCAGCCACGCGTTCCAGTG
ACCCCGGCACCGGCGGTGATTCCGATCCTGGTTATTGCCTGCGATCGTAGCACGGTTCGTCGTTGC
CTGGATAAACTGCTGCACTATCGCCCAAGCGCCGAACTGTTTCCGATTATCGTTAGCCAAGACTGT
GGCCATGAGGAGACGGCGCAAGCGATTGCAAGCTATGGCAGCGCCGTTACGCATATCCGCCAACCG
GATCTGAGCAGCATTGCAGTGCCGCCGGACCATCGCAAATTTCAGGGCTACTATAAGATCGCCCGT
CACTACCGCTGGGCACTGGGCCAGGTGTTCCGTCAATTTCGTTTTCCAGCCGCCGTGGTTGTGGAA
GACGATCTGGAAGTTGCCCCGGACTTTTTTGAGTATTTTCGTGCGACCTATCCACTGCTGAAAGCC
GATCCGAGCCTGTGGTGTGTGAGCGCATGGAACGACAATGGCAAAGAGCAGATGGTTGATGCGAGC
CGCCCAGAGCTGCTGTATCGTACCGATTTCTTTCCGGGCCTGGGTTGGCTGCTGCTGGCCGAGCTG
TGGGCGGAGCTGGAGCCGAAATGGCCGAAAGCATTCTGGGATGATTGGATGCGTCGTCCGGAACAA
CGCCAGGGTCGCGCCTGTATTCGCCCGGAGATTAGCCGTACCATGACCTTTGGTCGCAAAGGCGTG
AGCCACGGTCAATTCTTCGACCAGCACCTGAAGTTCATCAAGCTGAACCAACAGTTTGTGCATTTT
ACGCAGCTGGACCTGAGCTACCTGCAGCGCGAAGCGTATGATCGTGACTTTCTGGCACGTGTTTAT
GGTGCGCCGCAACTGCAAGTGGAGAAGGTTCGTACCAATGATCGCAAGGAACTGGGCGAAGTGCGT
GTGCAATATACCGGTCGTGACAGCTTTAAGGCGTTCGCAAAAGCCCTGGGCGTTATGGACGACCTG
AAAAGCGGCGTGCCACGTGCGGGTTATCGTGGTATTGTTACCTTCCAGTTTCGCGGTCGCCGCGTG
CATCTGGCCCCACCACTGACGTGGGAAGGTTATGATCCAAGCTGGAAT**CATCATCATCACCACCAC**
TAA

**(b)**

MKIEEGKLVIWINGDKGYNGLAEVGKKFEKDTGIKVTVEHPDKLEEKFPQVAATGDGPDIIFWAHD
RFGGYAQSGLLAEITPDKAFQDKLYPFTWDAVRYNGKLIAYPIAVEALSLIYNKDLLPNPPKTWEE
IPALDKELKAKGKSALMFNLQEPYFTWPLIAADGGYAFKYENGKYDIKDVGVDNAGAKAGLTFLVD
LIKNKHMNADTDYSIAEAAFNKGETAMTINGPWAWSNIDTSKVNYGVTVLPTFKGQPSKPFVGVLS
AGINAASPNKELAKEFLENYLLTDEGLEAVNKDKPLGAVALKSYEEELAKDPRIAATMENAQKGEI
MPNIPQMSAFWYAVRTAVINAASGRQTVDEALKDAQT**NSSSNNNNNNNNNNLGIEGRISEF**WTRPA
PGRPPSVSALDGDPASLTREVIRLAQDAEVELERQRGLLQQIGDALSSQRGRVPTAAPPAQPRVPV
TPAPAVIPILVIACDRSTVRRCLDKLLHYRPSAELFPIIVSQDCGHEETAQAIASYGSAVTHIRQP
DLSSIAVPPDHRKFQGYYKIARHYRWALGQVFRQFRFPAAVVVEDDLEVAPDFFEYFRATYPLLKA
DPSLWCVSAWNDNGKEQMVDASRPELLYRTDFFPGLGWLLLAELWAELEPKWPKAFWDDWMRRPEQ
RQGRACIRPEISRTMTFGRKGVSHGQFFDQHLKFIKLNQQFVHFTQLDLSYLQREAYDRDFLARVY
GAPQLQVEKVRTNDRKELGEVRVQYTGRDSFKAFAKALGVMDDLKSGVPRAGYRGIVTFQFRGRRV
HLAPPLTWEGYDPSWN**HHHHHH**

**Figure S2.** The DNA (**a**) and the amino acid (**b**) sequences of Δ24Bt3994-His$_6$. The sequences from the vector are underlined. The linker and the His$_6$-tag sequences are shown in bold.

**(a)**
ATGGAAATTGATTACACATCGTATGTAAATCCTTTTATTGGCACCGATTTCACAGGAAACACTTAT
CCCGGCGCACAAGCCCCCTTCGGTATGGTGCAGCTCAGCCCGGACAACGGACTTCCGGGATGGGAC
CGCATCTCCGGCTATTTTTATCCGGACAGCACCATCGCCGGCTTCAGCCATACCCACCTTTCGGGA
ACAGGTGCAGGCGACTTGTACGACATCTCTTTCATGCCGGTCACACTGCCCTACAAAGAGGCAGAA
GCGCCGCTGGGCATCTACTCCAAGTTTTCTCACGATGAAGAGAGCGCCTATGCAGGCTACTATCAA
GTACGTCTGAAAGACTACCATATCAATGTGGAACTGACCGCAACCGAACGTTGTGGCATCCAGCGA
TACACCTTCCCCAAAGCCGAAGCCGCCATCTTCCTGAATCTGAAGAAAGCCATGAACTGGGACTTC
ACAAACGATTCGCACATCGAAGTAGTGGACTCTGTGACGATTCAAGGATACCGTTACTCCGACGGC
TGGGCACGCGACCAGCGAATCTACTTCCGCACCCGCTTCTCGAAGCCTTTCGACAAGGTGGAACTG
GATACGACTGCCATTATCAAAGACAAGCAACACATCGGTACGGCTGTCATCGCACGTTTTGACTTC
CATACCGAAGAAGGAGAACAGATTCTCGTCAATACCGCCATTTCCGGCGTCAGCATGGAAGGCGCA
GCCAAGAACCTGCAAGCCGAAGTGCCCGAAAATGACTTCGACAAGTATCTGGCAGAAACAAAAGCA
AACTGGAACCGCCAACTGGGAAAGATCGAAGTCGAGGGTGACAATCAAGACGATAAAGTAAACTTT
TATACGGCTCTGTACCACTCGATGATCGCACCTACGATTTACAGTGACGTAGACGGAGCCTATTAC
GGTCCCGACAAAAAAGTACATCAGAGTGACGGCTGGGTGAATTACAGCACTTTCTCCCTTTGGGAT
ACCTACCGTGCCGCCCATCCCCTTTTCACATATACCGAACCGGAACGTGTCAACGACATGGTAAAA
TCCTTCATCGCCTTCTTTGAACAGAACGGACGCCTGCCCGTATGGAACTTCTACGGAGTGAAACC
GATATGATGATCGGCTATCATGCCGTTCCCGTGATTGTAGACGCTTATCTGAAAGGTATAGGCAAC
TTTGATGCGGAGAAAGCACTGGCCGCCTGCGTAGCTACCGCCAACTTGGATAACTACCGGGGCATC
GGGCTATACAAGCAATTAGGCTATATTCCTTACAACGTGACAGATCATTACAATGCCGAAAATTGG
TCACTGTCCAAAACACTGGAATATGCATTTGACGATTACTGCATCGCTGAGATGGCGAACAAGATG
GGCAAGAAGGAGATAGCGGACGAATTCTACAAACGTTCTCAGAACTACAAGAATGTTTACAACCCG
GCCACTTCGTTCATGCAGCCGCGTGACGATAAAGGAAACTTTATCAAAGACTTCAAAGCCGACGAA
TACACTCCGCATATCTGCGAAAGCAACGGCTGGCAATACTTCTGGTCCGTGCAGCATGACATAGAT
GGTCTGATTGATCTCACAGGAGGTAAAAACCGCTTTGCAGAGAAACTGGACAGCATGTTTACCTAC
CATCCGGCAGCCGACGAAGAACTTCCCATCTTCAGCACGGGAATGATCGGGCAATATGCTCACGGC
AATGAGCCGAGCCATCACGTAATCTATCTCTTCAATGCCGTAGGACAACAGAACCTGACACAGAAA
TACGTTGCCAAGGTAATGAACGAGCTTTACAAGAACGAGCCTGCCGGTCTTTGCGGAAACGAAGAC
TGCGGACAAATGTCTGCCTGGTACGTATTCAGCGCGATGGGATTCTATCCCGTCAATCCGGTCAGC
GGAAAGTATGAAATAGGTACTCCCCTGTTCCCGGAGATGAAGTTGCATCTGGCTAACGGAAAGACA
TTTACAGTACTTGCTCCCAAAGTGAGCAAAGAGAATATTTACATTCAATCTATCAAAGTAGACGGT
CAACCGTACAACAAGACCTACCTTACCCATGAACAGATTATGAGCGGTACCACCGTCGAATTTGAA
ATGGGGAATACTCCCTTAGTGGAAGTCGAATTTGAAGAACAGACCCAG**CTCGAGCACCACCACCAC**
**CACCAC**TGA

**(b)**
MEIDYTSYVNPFIGTDFTGNTYPGAQAPFGMVQLSPDNGLPGWDRISGYFYPDSTIAGFSHTHLSG
TGAGDLYDISFMPVTLPYKEAEAPLGIYSKFSHDEESAYAGYYQVRLKDYHINVELTATERCGIQR
YTFPKAEAAIFLNLKKAMNWDFTNDSHIEVVDSVTIQGYRYSDGWARDQRIYFRTRFSKPFDKVEL
DTTAIIKDKQHIGTAVIARFDFHTEEGEQILVNTAISGVSMEGAAKNLQAEVPENDFDKYLAETKA
NWNRQLGKIEVEGDNQDDKVNFYTALYHSMIAPTIYSDVDGAYYGPDKKVHQSDGWVNYSTFSLWD
TYRAAHPLFTYTEPERVNDMVKSFIAFFEQNGRLPVWNFYGSETDMMIGYHAVPVIVDAYLKGIGN
FDAEKALAACVATANLDNYRGIGLYKQLGYIPYNVTDHYNAENWSLSKTLEYAFDDYCIAEMANKM
GKKEIADEFYKRSQNYKNVYNPATSFMQPRDDKGNFIKDFKADEYTPHICESNGWQYFWSVQHDID
GLIDLTGGKNRFAEKLDSMFTYHPAADEELPIFSTGMIGQYAHGNEPSHHVIYLFNAVGQQNLTQK
YVAKVMNELYKNEPAGLCGNEDCGQMSAWYVFSAMGFYPVNPVSGKYEIGTPLFPEMKLHLANGKT
FTVLAPKVSKENIYIQSIKVDGQPYNKTYLTHEQIMSGTTVEFEMGNTPLVEVEFEEQTQ**LEHHHH**
**HH**

**Figure S3.** The DNA (**a**) and the amino acid (**b**) sequences of Δ18Bt1769-$His_6$. The sequences from the vector are underlined. The linker and the $His_6$-tag sequences are shown in bold.

**(a)**

ATGTGTAACGGTAGCCTGCAAACAGCGGATCGGACACCGGTCGATTACGTCAACCCGTATATCGGC
AACATCAGCCATCTGCTGGTTCCCACGTTCCCCACCATCCAGTTACCCAACAGTATGCTTCGTGTT
TACCCGGAACGCGCCGACTACACCTCCGAACTGCTGAAGGGGCTTCCATTGATTGTCACCAATCAC
CGGGAACGTTCCGCATTCAATTTCAGTCCCTATCAGGGAGAAAAGCTGCGTCCGGTCATAACGTAC
AACTATGACAACGAGCACATTACTCCTTATTCTTTCGATGTGGAACTGGATGACAACCGTATGAAA
GCGGAATATGCGCTTTCCCACCAGTCGGCTATCTACCGGATAACGTACGAGGCAGACAAACCGGCT
TACCTCATCGTAAACTCCCGCAATGGCTCTATCCACGCCAACGAGAACTTCATCAGCGGCCGCCAG
CAACTAAATGACAACACCAATGTCTATGTATATATAGAAGCACAGGAGAAGCCGATAAGCGCCGGC
ATCCTCGAAAACGGCACTATTGAAACAAGTAAGGATAATGCCGAAGGCGCAAACGCCTGCGCGGCA
TGGCGCTTTGCCGATGGAACCACAACGGTCAACCTCCGCTACGGTATTTCTTTTATCAGCGAGGAA
CAGGCCGAAAAGAACCTGCACCGCGAATTAAAAGACTATAACATCAAAGCACTGGCAGAAGCAGGA
CGCCAAATCTGGAACGAAACGTTGGGACGTATCCAAGTAGAAGGAGGAACGGAAGATGACAAAACC
GTATTCTACTCTTCTTTCTACCGTACCTTCGAACGCCCTATCTGCATGAGCGAAGGCGGCCGTTAC
TTCAGCGCCTTCGACGGAAAAGTACACGAAGACAACGGTACTCCTTTCTACACCGACGACTGGATT
TGGGACACTTACCGTGCCGCACATCCTCTCCGCACACTGATCGACCAACAAAAGGAAGAAGACATC
ATCGCATCCTACCTGCGCATGGCGGAACAAATGGGCAATATGTGGATGCCTACTTTCCCCGAAGTA
ACAGGTGACACACGCCGTATGAACTCCAATCATGCCGTTGCCACGGTAGCCGACGCACTGGCAAAA
GGGTTGAAAGTAGACACGGCAAAAGCCTACGAAGCTTGCCGGAAAGGGATTGAAGAAAAGACGCTC
GCCCCGTGGTCGGGAGCACCGGCCGGCTGGCTGGACAATTTCTACCGTGAAAACGGTTATATCCCC
GCCCTCCGTGTGGATGAACCGGAAAACGACCCGAACGTACATCCTTTCGAGAAGCGCCAGCCTGTC
GCTGTCACTCTGGGAACCAGCTACGACCAGTGGTGTCTCTCCCGTATCGCCCAAGCTCTGAACAAG
AAGGAAGAGGCCGAATACTATCTGAAATGTTCGTATAACTACCGGAATCTCTACAATAAAGAGACC
GCTTTCTTCCATCCGAAAGATAAGGAAGGACAATGGATTGAACCATTCGACTACCGTTTTCCCGGA
GGCATGGGAGCCCGTGAGTATTATGGAGAAAACAACGGCTGGGTTTACCGCTGGGACGTGCCACAC
AATGTAGCCGACCTCATCAGCCTTATGGGAGGAAACGAGCAGTTCATCGCCAACCTTGACCGTACT
TTCACCGAACCGCTGGGACGGAGCAAATATGCATTCTACGCGAAACTGCCCGACCACACGGGTAAT
GTAGGCCAATTCTCGATGGCGAACGAACCGTCTTTGCACGTTCCTTATCTTTATAACTACGCCGGA
CAGCCGTGGAAAACACAGAAGCGCATCCGTCAAATGCTGAAAACATGGTTCCGCAATGACCTGATG
GGTATTCCGGGAGATGAGGACGGCGGAGGTATGACTTCATTTGTGGTCTTCTCCTCCCTGGGCTTC
TACCCTGTCACTCCGGGATTGCCGGCCTACACTATCGGAAGTCCGCTTTTCACAGATGCAAAGATT
AGACTCAGCAACGGAGCTGTCTTCGAAATCGAAGCTAAGAACGCTTCCACTGATAACAAATACATC
CAGTCCGCCACGCTGAATGGCAAAGAATGGAACAAATCATGGTTCAGCCATGATGACCTGATGAGC
GGTGGCAAGCTAGTATTGGTGATGGGCAACAAACCCAACAAGACATGGGCCAGCGGAGCAGAGGAT
GTTCCTCCATCATTAGAAATCAAG**CTCGAGCACCACCACCACCACCAC**TGA

**(b)**

MCNGSLQTADRTPVDYVNPYIGNISHLLVPTFPTIQLPNSMLRVYPERADYTSELLKGLPLIVTNH
RERSAFNFSPYQGEKLRPVITYNYDNEHITPYSFDVELDDNRMKAEYALSHQSAIYRITYEADKPA
YLIVNSRNGSIHANENFISGRQQLNDNTNVYVYIEAQEKPISAGILENGTIETSKDNAEGANACAA
WRFADGTTTVNLRYGISFISEEQAEKNLHRELKDYNIKALAEAGRQIWNETLGRIQVEGGTEDDKT
VFYSSFYRTFERPICMSEGGRYFSAFDGKVHEDNGTPFYTDDWIWDTYRAAHPLRTLIDQQKEEDI
IASYLRMAEQMGNMWMPTFPEVTGDTRRMNSNHAVATVADALAKGLKVDTAKAYEACRKGIEEKTL
APWSGAPAGWLDNFYRENGYIPALRVDEPENDPNVHPFEKRQPVAVTLGTSYDQWCLSRIAQALNK
KEEAEYYLKCSYNYRNLYNKETAFFHPKDKEGQWIEPFDYRFPGGMGAREYYGENNGWVYRWDVPH
NVADLISLMGGNEQFIANLDRTFTEPLGRSKYAFYAKLPDHTGNVGQFSMANEPSLHVPYLYNYAG
QPWKTQKRIRQMLKTWFRNDLMGIPGDEDGGGMTSFVVFSSLGFYPVTPGLPAYTIGSPLFTDAKI
RLSNGAVFEIEAKNASTDNKYIQSATLNGKEWNKSWFSHDDLMSGGKLVLVMGNKPNKTWASGAED
VPPSLEIK**LEHHHHHH**

**Figure S4.** The DNA (**a**) and the amino acid (**b**) sequences of MBP-Δ27hGnT-II-$His_6$. The sequences from the vector are underlined. The linker the $His_6$-tag sequences are shown in bold.

**(a)**

ATGAAAATCGAAGAAGGTAAACTGGTAATCTGGATTAACGGCGATAAAGGCTATAACGGTCTCGCT
GAAGTCGGTAAGAAATTCGAGAAAGATACCGGAATTAAAGTCACCGTTGAGCATCCGGATAAACTG
GAAGAGAAATTCCCACAGGTTGCGGCAACTGGCGATGGCCCTGACATTATCTTCTGGGCACACGAC
CGCTTTGGTGGCTACGCTCAATCTGGCCTGTTGGCTGAAATCACCCCGGACAAAGCGTTCCAGGAC
AAGCTGTATCCGTTTACCTGGGATGCCGTACGTTACAACGGCAAGCTGATTGCTTACCCGATCGCT
GTTGAAGCGTTATCGCTGATTTATAACAAAGATCTGCTGCCGAACCCGCCAAAAACCTGGGAAGAG
ATCCCGGCGCTGGATAAAGAACTGAAAGCGAAAGGTAAGAGCGCGCTGATGTTCAACCTGCAAGAA
CCGTACTTCACCTGGCCGCTGATTGCTGCTGACGGGGGTTATGCGTTCAAGTATGAAAACGGCAAG
TACGACATTAAAGACGTGGGCGTGGATAACGCTGGCGCGAAAGCGGGTCTGACCTTCCTGGTTGAC
CTGATTAAAAACAAACACATGAATGCAGACACCGATTACTCCATCGCAGAAGCTGCCTTTAATAAA
GGCGAAACAGCGATGACCATCAACGGCCCGTGGGCATGGTCCAACATCGACACCAGCAAAGTGAAT
TATGGTGTAACGGTACTGCCGACCTTCAAGGGTCAACCATCCAAACCGTTCGTTGGCGTGCTGAGC
GCAGGTATTAACGCCGCCAGTCCGAACAAAGAGCTGGCAAAAGAGTTCCTCGAAAACTATCTGCTG
ACTGATGAAGGTCTGGAAGCGGTTAATAAAGACAAACCGCTGGGTGCCGTAGCGCTGAAGTCTTAC
GAGGAAGAGTTGGTGAAAGATCCGCGGATTGCCGCCACTATGGAAAACGCCCAGAAAGGTGAAATC
ATGCCGAACATCCCGCAGATGTCCGCTTTCTGGTATGCCGTGCGTACTGCGGTGATCAACGCCGCC
AGCGGTCGTCAGACTGTCGATGAAGCCCTGAAAGACGCGCAGACT**AATTCGAGCTCGAACAACAAC**
**AACAATAACAATAACAACAACCTCGGGATCGAGGGAAGGATTTCAGAATTCGGATCC**AACGGTCGT
CAGCGTAAAAACGAGGCGCTGGCACCGCCGCTGCTGGACGCTGAACCGGCTCGTGGTGCTGGCGGT
CGCGGTGGCGACCATCCGTCTGTGGCAGTTGGTATCCGTCGTGTTTCCAACGTGAGCGCTGCGAGC
CTGGTGCCGGCTGTGCCGCAGCCGGAAGCGGATAACCTGACTCTGCGCTATCGCTCTCTGGTTTAC
CAGCTGAACTTTGATCAGACTCTGCGTAACGTTGACAAAGCGGGTACCTGGGCTCCGCGCGAACTG
GTTCTGGTGGTTCAGGTTCACAACCGTCCGGAGTATCTGCGTCTGCTGCTGGATAGCCTGCGCAAA
GCGCAGGGTATTGATAACGTTCTGGTTATCTTTTCTCATGACTTCTGGTCTACCGAGATCAACCAG
CTGATTGCAGGTGTTAACTTCTGCCCGGTGCTGCAGGTTTTCTTCCCGTTCTCCATCCAGCTGTAC
CCGAACGAATTCCCGGGTTCCGATCCGCGTGATTGCCCGCGTGACCTGCCGAAGAACGCGGCTCTG
AAGCTGGGTTGCATCAACGCGGAATACCCGGACTCTTTCGGTCACTACCGTGAAGCGAAATTCTCT
CAGACCAAACACCATTGGTGGTGGAAACTGCATTTCGTTTGGGAACGTGTTAAAATCCTGCGCGAC
TACGCGGGTCTGATTCTGTTCCTGGAAGAGGACCACTACCTGGCTCCGGATTTCTACCACGTTTTC
AAAAAGATGTGGAAGCTGAAACAGCAGGAATGCCCGGAATGCGATGTTCTGTCCCTGGGTACTTAT
AGCGCGAGCCGTTCCTTTTATGGCATGGCGGACAAAGTTGACGTTAAAACTTGGAAAAGCACCGAA
CACAACATGGGTCTGGCACTGACTCGCAACGCATATCAGAAACTGATCGAATGCACCGACACCTTT
TGCACCTACGACGACTACAACTGGGATTGGACCCTGCAGTACCTGACCGTTTCTTGTCTGCCGAAA
TTTTGGAAAGTGCTGGTTCCGCAGATCCCGCGCATCTTCCATGCGGGTGACTGCGGTATGCATCAC
AAGAAGACTTGTCGTCCGTCTACCCAGAGCGCGCAGATTGAATCTCTGCTGAACAACAACAAACAG
TATATGTTCCCGGAAACTCTGACCATCTCTGAAAAATTCACCGTGGTTGCGATCTCTCCGCCGCGT
AAGAACGGTGGTTGGGGCGACATTCGTGACCACGAACTGTGCAAATCCTATCGCCGCCTGCAG**CAC**
**CACCACCACCACCAC**TAA

**(b)**

MKIEEGKLVIWINGDKGYNGLAEVGKKFEKDTGIKVTVEHPDKLEEKFPQVAATGDGPDIIFWAHD
RFGGYAQSGLLAEITPDKAFQDKLYPFTWDAVRYNGKLIAYPIAVEALSLIYNKDLLPNPPKTWEE
IPALDKELKAKGKSALMFNLQEPYFTWPLIAADGGYAFKYENGKYDIKDVGVDNAGAKAGLTFLVD
LIKNKHMNADTDYSIAEAAFNKGETAMTINGPWAWSNIDTSKVNYGVTVLPTFKGQPSKPFVGVLS
AGINAASPNKELAKEFLENYLLTDEGLEAVNKDKPLGAVALKSYEEELVKDPRIAATMENAQKGEI
MPNIPQMSAFWYAVRTAVINAASGRQTVDEALKDAQT**NSSSNNNNNNNNNNLGIEGRISEFGS**NGR
QRKNEALAPPLLDAEPARGAGGRGGDHPSVAVGIRRVSNVSAASLVPAVPQPEADNLTLRYRSLVY
QLNFDQTLRNVDKAGTWAPRELVLVVQVHNRPEYLRLLLDSLRKAQGIDNVLVIFSHDFWSTEINQ
LIAGVNFCPVLQVFFPFSIQLYPNEFPGSDPRDCPRDLPKNAALKLGCINAEYPDSFGHYREAKFS
QTKHHWWWKLHFVWERVKILRDYAGLILFLEEDHYLAPDFYHVFKKMWKLKQQECPECDVLSLGTY
SASRSFYGMADKVDVKTWKSTEHNMGLALTRNAYQKLIECTDTFCTYDDYNWDWTLQYLTVSCLPK
FWKVLVPQIPRIFHAGDCGMHHKKTCRPSTQSAQIESLLNNNKQYMFPETLTISEKFTVVAISPPR
KNGGWGDIRDHELCKSYRRLQ**HHHHHH**

**Figure S5.** The DNA (**a**) and the amino acid (**b**) sequences of MBP-Δ128Bβ4GalT1-$His_6$. The sequences from the vector are underlined. The linker and the $His_6$-tag sequences are shown in bold.

**(a)**
ATGAAAATCGAAGAAGGTAAACTGGTAATCTGGATTAACGGCGATAAAGGCTATAACGGTCTCGCT
GAAGTCGGTAAGAAATTCGAGAAAGATACCGGAATTAAAGTCACCGTTGAGCATCCGGATAAACTG
GAAGAGAAATTCCCACAGGTTGCGGCAACTGGCGATGGCCCTGACATTATCTTCTGGGCACACGAC
CGCTTTGGTGGCTACGCTCAATCTGGCCTGTTGGCTGAAATCACCCCGGACAAAGCGTTCCAGGAC
AAGCTGTATCCGTTTACCTGGGATGCCGTACGTTACAACGGCAAGCTGATTGCTTACCCGATCGCT
GTTGAAGCGTTATCGCTGATTTATAACAAAGATCTGCTGCCGAACCCGCCAAAAACCTGGGAAGAG
ATCCCGGCGCTGGATAAAGAACTGAAAGCGAAAGGTAAGAGCGCGCTGATGTTCAACCTGCAAGAA
CCGTACTTCACCTGGCCGCTGATTGCTGCTGACGGGGGTTATGCGTTCAAGTATGAAAACGGCAAG
TACGACATTAAAGACGTGGGCGTGGATAACGCTGGCGCGAAAGCGGGTCTGACCTTCCTGGTTGAC
CTGATTAAAAACAAACACATGAATGCAGACACCGATTACTCCATCGCAGAAGCTGCCTTTAATAAA
GGCGAAACAGCGATGACCATCAACGGCCCGTGGGCATGGTCCAACATCGACACCAGCAAAGTGAAT
TATGGTGTAACGGTACTGCCGACCTTCAAGGGTCAACCATCCAAACCGTTCGTTGGCGTGCTGAGC
GCAGGTATTAACGCCGCCAGTCCGAACAAAGAGCTGGCAAAAGAGTTCCTCGAAAACTATCTGCTG
ACTGATGAAGGTCTGGAAGCGGTTAATAAAGACAAACCGCTGGGTGCCGTAGCGCTGAAGTCTTAC
GAGGAAGAGTTGGCGAAAGATCCACGTATTGCCGCCACTATGGAAAACGCCCAGAAAGGTGAAATC
ATGCCGAACATCCCGCAGATGTCCGCTTTCTGGTATGCCGTGCGTACTGCGGTGATCAACGCCGCC
AGCGGTCGTCAGACTGTCGATGAAGCCCTGAAAGACGCGCAGACT**AATTCGAGCTCGAACAACAAC**
**AACAATAACAATAACAACAACCTCGGGATCGAGGGAAGGATTTCAGAATTC**CGCTCGCTGACCGCA
TGCCCTGAGGAGTCCCCGCTGCTCGTCGGCCCCATGCTGATTGAGTTTAACATACCTGTGGACCTG
AAGCTTGTGGAGCAGCAGAACCCGAAGGTGAAGTTGGGTGGTCGCTACACCCCCATGGACTGCATC
TCTCCTCACAAGGTGGCCATCATCATTCCATTCCGCAACCGGCAGGAACACCTCAAGTACTGGCTG
TATTACTTGCACCCAATCCTACAGCGTCAGCAGTTAGACTATGGCATCTATGTTATCAACCAGGCT
GGAGAGTCCATGTTCAACCGCGCAAAGCTCCTCAATGTTGGCTTTAAAGAGGCCTTGAAGGACTAT
GACTACAACTGCTTTGTGTTTAGCGATGTGGACCTCATCCCAATGAACGACCATAACACCTACAGG
TGCTTTTCACAGCCACGGCACATTTCTGTAGCAATGGATAAGTTTGGACTTAGCCTACCTTACGTG
CAGTATTTTGGAGGTGTCTCTGCTCTAAGTAAACAACAGTTTCTCAGCATCAATGGATTTCCTAAT
AACTACTGGGGCTGGGGAGGTGAAGATGATGACATTTATAACAGATTAGCTTTTAGAGGCATGTCT
GTGTCTCGCCCAAATGCTGTGATCGGGAAGTGTCGGATGATCCGCCACTCGAGAGACAAGAAAAAT
GAACCTAATCCTCAGAGGTTTGACCGAATTGCACATACAAAGGAGACAATGCTCTCTGATGGTTTG
AACTCACTCACCTACATGGTGTTAGAGGTCCAGAGGTACCCGTTGTATACCAAAATCACAGTGGAC
ATCGGGACGCCGAGC**CACCATCACCATCACCAT**TGA

**(b)**
MKIEEGKLVIWINGDKGYNGLAEVGKKFEKDTGIKVTVEHPDKLEEKFPQVAATGDGPDIIFWAHD
RFGGYAQSGLLAEITPDKAFQDKLYPFTWDAVRYNGKLIAYPIAVEALSLIYNKDLLPNPPKTWEE
IPALDKELKAKGKSALMFNLQEPYFTWPLIAADGGYAFKYENGKYDIKDVGVDNAGAKAGLTFLVD
LIKNKHMNADTDYSIAEAAFNKGETAMTINGPWAWSNIDTSKVNYGVTVLPTFKGQPSKPFVGVLS
AGINAASPNKELAKEFLENYLLTDEGLEAVNKDKPLGAVALKSYEEELAKDPRIAATMENAQKGEI
MPNIPQMSAFWYAVRTAVINAASGRQTVDEALKDAQT**NSSSNNNNNNNNNNLGIEGRISEF**RSLTA
CPEESPLLVGPMLIEFNIPVDLKLVEQQNPKVKLGGRYTPMDCISPHKVAIIIPFRNRQEHLKYWL
YYLHPILQRQQLDYGIYVINQAGESMFNRAKLLNVGFKEALKDYDYNCFVFSDVDLIPMNDHNTYR
CFSQPRHISVAMDKFGLSLPYVQYFGGVSALSKQQFLSINGFPNNYWGWGGEDDDIYNRLAFRGMS
VSRPNAVIGKCRMIRHSRDKKNEPNPQRFDRIAHTKETMLSDGLNSLTYMVLEVQRYPLYTKITVD
IGTPS**HHHHHH**

**Figure S6.** The DNA (**a**) and the amino acid (**b**) sequences of MBP-Δ89hST6GAL-I-$His_6$. The sequences from the vector are underlined. The linker and the $His_6$-tag sequences are shown in bold.

**(a)**

ATGAAAATCGAAGAAGGTAAACTGGTAATCTGGATTAACGGCGATAAAGGCTATAACGGTCTCGCT
GAAGTCGGTAAGAAATTCGAGAAAGATACCGGAATTAAAGTCACCGTTGAGCATCCGGATAAACTG
GAAGAGAAATTCCCACAGGTTGCGGCAACTGGCGATGGCCCTGACATTATCTTCTGGGCACACGAC
CGCTTTGGTGGCTACGCTCAATCTGGCCTGTTGGCTGAAATCACCCCGGACAAAGCGTTCCAGGAC
AAGCTGTATCCGTTTACCTGGGATGCCGTACGTTACAACGGCAAGCTGATTGCTTACCCGATCGCT
GTTGAAGCGTTATCGCTGATTTATAACAAAGATCTGCTGCCGAACCCGCCAAAAACCTGGGAAGAG
ATCCCGGCGCTGGATAAAGAACTGAAAGCGAAAGGTAAGAGCGCGCTGATGTTCAACCTGCAAGAA
CCGTACTTCACCTGGCCGCTGATTGCTGCTGACGGGGGTTATGCGTTCAAGTATGAAAACGGCAAG
TACGACATTAAAGACGTGGGCGTGGATAACGCTGGCGCGAAAGCGGGTCTGACCTTCCTGGTTGAC
CTGATTAAAAACAAACACATGAATGCAGACACCGATTACTCCATCGCAGAAGCTGCCTTTAATAAA
GGCGAAACAGCGATGACCATCAACGGCCCGTGGGCATGGTCCAACATCGACACCAGCAAAGTGAAT
TATGGTGTAACGGTACTGCCGACCTTCAAGGGTCAACCATCCAAACCGTTCGTTGGCGTGCTGAGC
GCAGGTATTAACGCCGCCAGTCCGAACAAAGAGCTGGCAAAAGAGTTCCTCGAAAACTATCTGCTG
ACTGATGAAGGTCTGGAAGCGGTTAATAAAGACAAACCGCTGGGTGCCGTAGCGCTGAAGTCTTAC
GAGGAAGAGTTGGCGAAAGATCCACGTATTGCCGCCACTATGGAAAACGCCCAGAAAGGTGAAATC
ATGCCGAACATCCCGCAGATGTCCGCTTTCTGGTATGCCGTGCGTACTGCGGTGATCAACGCCGCC
AGCGGTCGTCAGACTGTCGATGAAGCCCTGAAAGACGCGCAGACT**AATTCGAGCTCGAACAACAAC**
**AACAATAACAATAACAACAACCTCGGGATCGAGGGAAGGATTTCAGAATTC**GAGGCCTCGTTTCAG
GTTTGGAACAAGGACTCGAGTAGCAAGAATCTAATACCACGGCTGCAAAAGATATGGAAGAACTAT
CTAAGTATGAACAAGTATAAAGTGTCATATAAGGGACCGGGTCCAGGAATCAAATTTTCAGCCGAG
GCCCTTAGGTGCCACTTGCGAGACCATGTGAATGTGAGCATGGTCGAGGTGACAGACTTCCCTTTC
AACACGTCCGAGTGGGAAGGATATCTGCCCAAGGAATCGATCCGCACTAAGGCTGGTCCCTGGGGA
CGGTGTGCCGTCGTGTCGTCCGCCGGATCGTTGAAGTCGAGTCAATTGGGACGGGAGATAGACGAC
CATGACGCAGTCCTCAGGTTTAACGGCGCCCCGACGGCCAATTTCCAACAAGACGTAGGCACGAAA
ACAACCATCCGCCTCATGAATAGCCAACTGGTCACCACAGAGAAGCGATTCTTGAAGGACTCTCTG
TACAACGAGGGTATTTTGATTGTCTGGGACCCGTCCGTATATCACTCGGACATCCCGAAGTGGTAC
CAAAACCCCGATTACAATTTCTTCAATAACTATAAAACTTACCGCAAACTGCATCCGAACCAACCC
TTTTATATACTGAAGCCACAAATGCCGTGGGAACTGTGGGACATCTTGCAAGAAATAAGCCCCGAG
GAGATCCAACCAAATCCGCCATCTAGCGGCATGTTGGGCATCATAATAATGATGACTTTGTGTGAC
CAAGTTGACATCTATGAGTTCCTTCCATCCAAGCGCAAAACCGACGTCTGCTACTATTACCAAAAG
TTCTTTGACTCCGCCTGTACTATGGGAGCCTATCATCCGTTGTTGTACGAGAAGAACCTCGTCAAG
CATCTGAATCAGGGCACTGATGAAGATATCTACCTTCTTGGTAAAGCCACCCTACCGGGATTCCGA
ACAATACACTGC**CATCATCATCACCACCAC**TAA

**(b)**

MKIEEGKLVIWINGDKGYNGLAEVGKKFEKDTGIKVTVEHPDKLEEKFPQVAATGDGPDIIFWAHD
RFGGYAQSGLLAEITPDKAFQDKLYPFTWDAVRYNGKLIAYPIAVEALSLIYNKDLLPNPPKTWEE
IPALDKELKAKGKSALMFNLQEPYFTWPLIAADGGYAFKYENGKYDIKDVGVDNAGAKAGLTFLVD
LIKNKHMNADTDYSIAEAAFNKGETAMTINGPWAWSNIDTSKVNYGVTVLPTFKGQPSKPFVGVLS
AGINAASPNKELAKEFLENYLLTDEGLEAVNKDKPLGAVALKSYEEELAKDPRIAATMENAQKGEI
MPNIPQMSAFWYAVRTAVINAASGRQTVDEALKDAQT**NSSSNNNNNNNNNNLGIEGRISEF**EASFQ
VWNKDSSSKNLIPRLQKIWKNYLSMNKYKVSYKGPGPGIKFSAEALRCHLRDHVNVSMVEVTDFPF
NTSEWEGYLPKESIRTKAGPWGRCAVVSSAGSLKSSQLGREIDDHDAVLRFNGAPTANFQQDVGTK
TTIRLMNSQLVTTEKRFLKDSLYNEGILIVWDPSVYHSDIPKWYQNPDYNFFNNYKTYRKLHPNQP
FYILKPQMPWELWDILQEISPEEIQPNPPSSGMLGIIIMMTLCDQVDIYEFLPSKRKTDVCYYYQK
FFDSACTMGAYHPLLYEKNLVKHLNQGTDEDIYLLGKATLPGFRTIHC**HHHHHH**

**Figure S7.** The DNA (**a**) and the amino acid (**b**) sequences of MBP-CjCst-IΔ145-His$_6$. The sequences from the vector are underlined. The linker and the His$_6$-tag sequences are shown in bold.

**(a)**

ATGAAAATCGAAGAAGGTAAACTGGTAATCTGGATTAACGGCGATAAAGGCTATAACGGTCTCGCT
GAAGTCGGTAAGAAATTCGAGAAAGATACCGGAATTAAAGTCACCGTTGAGCATCCGGATAAACTG
GAAGAGAAATTCCCACAGGTTGCGGCAACTGGCGATGGCCCTGACATTATCTTCTGGGCACACGAC
CGCTTTGGTGGCTACGCTCAATCTGGCCTGTTGGCTGAAATCACCCCGGACAAAGCGTTCCAGGAC
AAGCTGTATCCGTTTACCTGGGATGCCGTACGTTACAACGGCAAGCTGATTGCTTACCCGATCGCT
GTTGAAGCGTTATCGCTGATTTATAACAAAGATCTGCTGCCGAACCCGCCAAAAACCTGGGAAGAG
ATCCCGGCGCTGGATAAAGAACTGAAAGCGAAAGGTAAGAGCGCGCTGATGTTCAACCTGCAAGAA
CCGTACTTCACCTGGCCGCTGATTGCTGCTGACGGGGGTTATGCGTTCAAGTATGAAAACGGCAAG
TACGACATTAAAGACGTGGGCGTGGATAACGCTGGCGCGAAAGCGGGTCTGACCTTCCTGGTTGAC
CTGATTAAAAACAAACACATGAATGCAGACACCGATTACTCCATCGCAGAAGCTGCCTTTAATAAA
GGCGAAACAGCGATGACCATCAACGGCCCGTGGGCATGGTCCAACATCGACACCAGCAAAGTGAAT
TATGGTGTAACGGTACTGCCGACCTTCAAGGGTCAACCATCCAAACCGTTCGTTGGCGTGCTGAGC
GCAGGTATTAACGCCGCCAGTCCGAACAAAGAGCTGGCAAAAGAGTTCCTCGAAAACTATCTGCTG
ACTGATGAAGGTCTGGAAGCGGTTAATAAAGACAAACCGCTGGGTGCCGTAGCGCTGAAGTCTTAC
GAGGAAGAGTTGGCGAAAGATCCACGTATTGCCGCCACTATGGAAAACGCCCAGAAAGGTGAAATC
ATGCCGAACATCCCGCAGATGTCCGCTTTCTGGTATGCCGTGCGTACTGCGGTGATCAACGCCGCC
AGCGGTCGTCAGACTGTCGATGAAGCCCTGAAAGACGCGCAGACT**AATTCGAGCTCGAACAACAAC**
**AACAATAACAATAACAACAACCTCGGGATCGAGGGAAGGATTTCAGAATTC**ATGACACGCACCAGA
ATGGAGAACGAGTTGATTGTCTCAAAGAACATGCAAAACATCATTATCGCAGGCAATGGCCCCAGC
CTCAAGAATATTAATTACAAGCGTCTCCCGCGGGAATACGACGTTTTCCGTTGTAACCAATTTTAC
TTCGAGGACAAGTATTACTTAGGGAAGAAGATAAAGGCAGTTTTCTTTAACCCTGGCGTATTCTTA
CAACAATACCACACCGCTAAGCAACTTATACTCAAGAATGAATACGAGATCAAGAACATTTTCTGT
TCCACTTTCAATCTCCCGTTTATTGAGAGCAACGATTTCCTGCACCAATTTTACAACTTCTTCCCC
GATGCAAAACTGGGCTACGAGGTTATCGAGAACCTGAAGGAATTTTACGCCTACATCAAGTATAAT
GAGATATACTTTAACAAGCGTATCACATCCGGGGTATATATGTGTGCCATCGCCATAGCCCTGGGT
TACAAGACCATTTACTTGTGCGGAATTGACTTCTACGAGGGCGATGTGATCTATCCATTCGAGGCT
ATGTCAACTAATATTAAGACCATCTTCCCGGGCATTAAAGACTTTAAGCCGAGCAATTGTCATAGT
AAAGAGTACGACATCGAAGCATTAAAACTTCTGAAATCGATATACAAGGTGAATATATACGCGCTC
TGTGACGACTCCATATTAGCAAACCATTTCCCTTTGAGCATCAACATCAATAACAATTTCACATTG
GAGAACAAGCACAACAATAGTATCAACGACATCCTGTTAACTGACAACACACCGGGTGTTTCATTT
TACAAGAATCAACTGAAAGCAGATAACAAGATAATGCTGAACTTTTATCAT**CATCATCATCATCAT**
TAA

**(b)**

MKIEEGKLVIWINGDKGYNGLAEVGKKFEKDTGIKVTVEHPDKLEEKFPQVAATGDGPDIIFWAHD
RFGGYAQSGLLAEITPDKAFQDKLYPFTWDAVRYNGKLIAYPIAVEALSLIYNKDLLPNPPKTWEE
IPALDKELKAKGKSALMFNLQEPYFTWPLIAADGGYAFKYENGKYDIKDVGVDNAGAKAGLTFLVD
LIKNKHMNADTDYSIAEAAFNKGETAMTINGPWAWSNIDTSKVNYGVTVLPTFKGQPSKPFVGVLS
AGINAASPNKELAKEFLENYLLTDEGLEAVNKDKPLGAVALKSYEEELAKDPRIAATMENAQKGEI
MPNIPQMSAFWYAVRTAVINAASGRQTVDEALKDAQT**NSSSNNNNNNNNNNLGIEGRISEF**MTRTR
MENELIVSKNMQNIIIAGNGPSLKNINYKRLPREYDVFRCNQFYFEDKYYLGKKIKAVFFNPGVFL
QQYHTAKQLILKNEYEIKNIFCSTFNLPFIESNDFLHQFYNFFPDAKLGYEVIENLKEFYAYIKYN
EIYFNKRITSGVYMCAIAIALGYKTIYLCGIDFYEGDVIYPFEAMSTNIKTIFPGIKDFKPSNCHS
KEYDIEALKLLKSIYKVNIYALCDDSILANHFPLSININNNFTLENKHNNSINDILLTDNTPGVSF
YKNQLKADNKIMLNFY**HHHHHH**
